# Body fluid multiomics in 3PM-guided ischemic stroke management: health risk assessment, targeted protection against health-to-disease transition, and cost-effective personalized approach are envisaged

**DOI:** 10.1007/s13167-024-00376-2

**Published:** 2024-08-29

**Authors:** Ruofei Chen, Xiaoyan Wang, Na Li, Olga Golubnitschaja, Xianquan Zhan

**Affiliations:** 1 Shandong Provincial Key Laboratory of Precision Oncology, Shandong Cancer Hospital and Institute, Shandong First Medical University & Shandong Academy of Medical Sciences, 440 Jiyan Road, Jinan, Shandong 250117 P. R. China; 2 Predictive, Preventive and Personalised (3P) Medicine, University Hospital Bonn, Venusberg Campus 1, Rheinische Friedrich-Wilhelms-University of Bonn, Bonn, 53127 Germany; 3 Shandong Provincial Key Medical and Health Laboratory of Ovarian Cancer Multiomics, & Jinan Key Laboratory of Cancer Multiomics, Shandong First Medical University & Shandong Academy of Medical Sciences, 6699 Qingdao Road, Jinan, Shandong 250117 P. R. China

**Keywords:** Predictive preventive personalized medicine (PPPM / 3PM), Ischemic stroke, Suboptimal health, Lifestyle, Modifiable risk factors, Health-to-disease transition, Primary and secondary care, Patient-friendly non-invasive approach, Multiomics, Mitochondrial stress and homeostasis, Diabetes mellitus, Diabetic retinopathy, Flammer syndrome, Health risk assessment, Individualized patient profile, Artificial intelligence, Population screening, Healthcare economy, Health policy, Expert recommendations

## Abstract

Because of its rapid progression and frequently poor prognosis, stroke is the third major cause of death in Europe and the first one in China. Many independent studies demonstrated sufficient space for prevention interventions in the primary care of ischemic stroke defined as the most cost-effective protection of vulnerable subpopulations against health-to-disease transition. Although several studies identified molecular patterns specific for IS in body fluids, none of these approaches has yet been incorporated into IS treatment guidelines. The advantages and disadvantages of individual body fluids are thoroughly analyzed throughout the paper. For example, multiomics based on a minimally invasive approach utilizing blood and its components is recommended for real-time monitoring, due to the particularly high level of dynamics of the blood as a body system. On the other hand, tear fluid as a more stable system is recommended for a non-invasive and patient-friendly holistic approach appropriate for health risk assessment and innovative screening programs in cost-effective IS management. This article details aspects essential to promote the practical implementation of highlighted achievements in 3PM-guided IS management.

**Supplementary Information:**

The online version contains supplementary material available at 10.1007/s13167-024-00376-2.

## Introduction

Because of its rapid progression and frequently poor prognosis, stroke is the third major factor of death in Europe and the first in China [1]. Over the past 30 years, the impact of stroke has been increasing in many areas of China, especially in rural areas, with a gradient of incidence between north and south and the heaviest load of stroke in the northern and central parts of China [2]. According to the pathogenesis of stroke, stroke is categorized into hemorrhagic stroke (HS) and ischemic stroke (IS). HS accounts for 13%, whereas IS represents the main type of morbidity accounting for 87% of all stroke cases [3]. The main mechanism of IS is the narrowing or blockage of blood vessels that transport oxygen and provide nutrients for the cerebral tissue. The most effective clinically available therapy for acute ischemic stroke (AIS) is reperfusion therapy. In addition to intravenous (IV) thrombolysis with the application of FDA-approved rtPA (recombinant tissue-type plasminogen activator) within the defined timeframe of stroke occurrence (4.5 h) and endovascular treatment of patients with large-artery occlusion who undergoing mechanical thrombectomy (6 h) [4], CT perfusion or magnetic resonance (MR) diffusion/perfusion advanced brain imaging (penumbral imaging) can also be used for intracerebral risk visualization [5], thus evaluating the patient’s risk on basis of ASPECTS in connection with the Rapid Arterial Occlusion Evaluation/NIHSS Score and expanding the time window for thrombolysis (9–24 h) on top of receiving endovascular therapy [6], increasing the chances of patient salvage. However, reperfusion therapy for AIS is associated with significant health risks. When there are problems with time window selection and patient candidacy, ischemia–reperfusion injury occurs after reperfusion with intravenous thrombolysis and mechanical thrombectomy, which is mainly manifested as hemorrhage and edema, jeopardizing patient prognosis and life [4]. In addition, numerous contraindications to thrombolytic therapy, such as previous head injury, intracranial hemorrhage, suspected infective endocarditis, and aortic arch entrapment, impede the implementation of reperfusion therapies. Although intravenous (IV) thrombolytic therapy with rtPA is the only therapy approved for AIS, the utilization rate is reported to be lower than 6% of all patients in the USA [7]. The limitations of existing clinical treatments have raised the demand for accurate diagnosis, good prediction, and improved prognosis of IS. Predictive, preventive, and personalized medicine (PPPM; 3PM) plays a key role in resolving the demand.

## Evidence-based working hypotheses in the framework of 3PM

*Hypothesis A: multi‑factorial systemic risks of IS are preventable making a holistic 3PM approach in primary care to the most cost-effective IS management.*

**Fig. 1:**
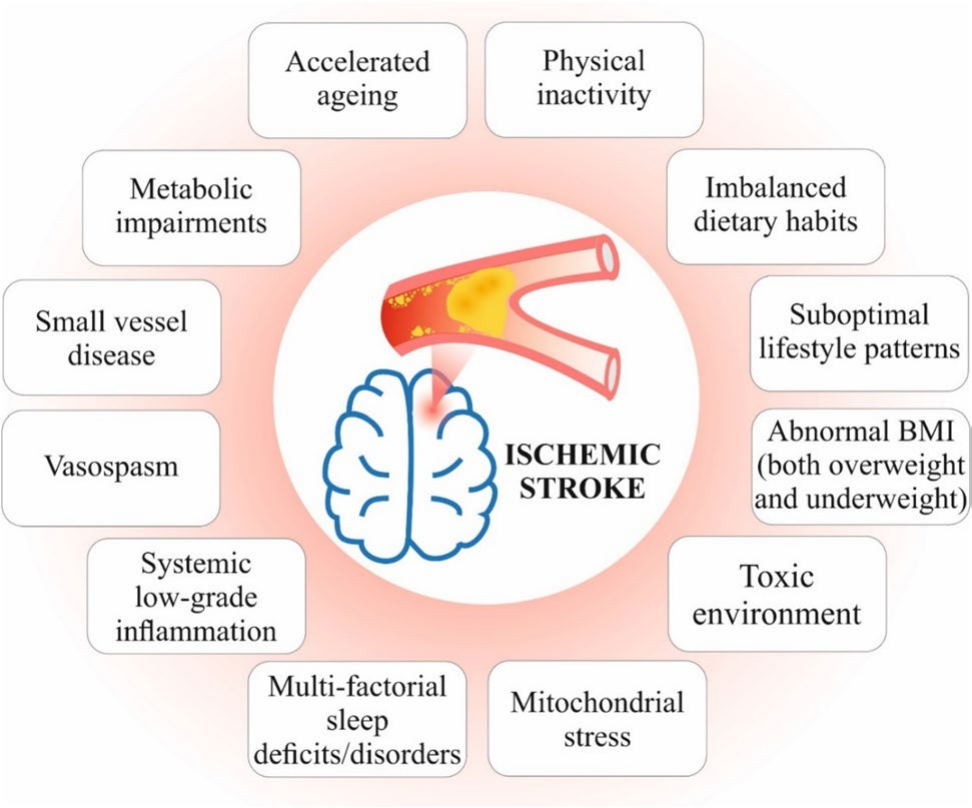
IS as a multifactorial disease—a number of risks are preventable within primary care; the image and contents are adapted from [11, 17]

Many independent studies demonstrated sufficient space for prevention interventions in IS primary care defined as the most cost-effective protection of vulnerable subpopulations against health-to-disease transition [8–11]. Clearly identified for a holistic 3PM approach, preventable systemic risks are summarized in Fig. 1. All risk factors are interrelated at molecular and organismal levels such as mitochondrial stress caused by toxic environment and suboptimal lifestyle including dietary and psychosocial patterns, etc., which in turn lead to disturbed microcirculation, systemic inflammation, metabolic impairments, small-vessel disease, vascular aging, inflammaging, and clinical development of IS [11–16]. Here, we hypothesize that a holistic 3PM approach implemented within primary care is feasible as the most cost-effective individualized protection against health-to-disease transition.

*Hypothesis B: body fluid molecular patterns are instrumental for holistic 3PM-guided population screening, health risk assessment, targeted prevention, and therapy monitoring in primary and secondary care of IS.*

This evidence-based hypothesis can be exemplified on research data recently presented by international IS-relevant projects utilizing tear fluid molecular patterns for.

**Fig. 2:**
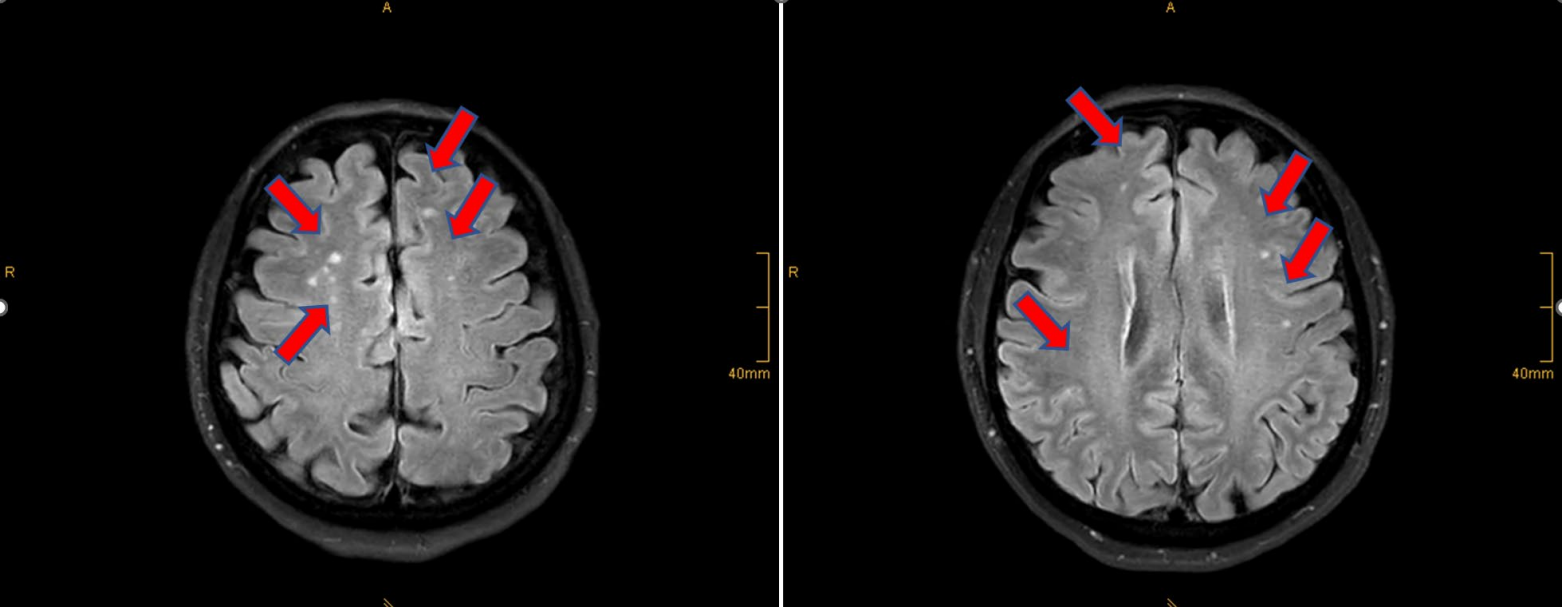
Brain MRI of an individual with the Flammer syndrome phenotype; the marked multi-site lacunar microinfarction is asymptomatic; the image is adapted from [11]

1. *Primary care* focused on suboptimal health of the Flammer syndrome phenotype (FSP) carriers, who are strongly predisposed to silent lacunar ischemic brain lesions (asymptomatic IS) (see Fig. 2); FSP is very well detailed in the scientific literature [18, 19] being therefore suitable for population screening—the specialized survey is available; moreover, FSP individuals develop their phenotype early in life, usually during puberty, that is highly relevant for the cost-effective 3PM implementation in primary care [14, 19]; tear fluid molecular pattern analysis performed in FSP individuals demonstrates significant alterations in mitochondrial health and homeostasis reflected in mitophagy, which is herewith hypothesized as a potent target for predictive diagnostics and individualized protection against health-to-disease transition in FSP-affected individuals [20, 21].
2. *Secondary care* can be exemplified on patients diagnosed with diabetes mellitus (DM); DM patients are generally at increased risk of IS; however, an accurate patient stratification is essential for individualized IS prediction and patient stratification in the DM cohort; an early indicator of the IS risk is a development of proliferative diabetic retinopathy (PDR) [17]; recently published research data have demonstrated metabolic patterns in the tear fluid shared by both IS and PDR patient cohorts [11, 22].

## Weighting the role of individual body fluids in 3PM-guided IS management

IS is a multifactorial cerebrovascular illness with an extremely complex pathophysiology [23] resulting from damaged blood flow supply systems and involving the development of ischemic necrosis of core vascular beds and tissues as well as damage to the peripheral ischemic hemi-diaphragm [24–26]. The pathophysiological mechanisms of ischemic brain injury encompass the activation of a series of deleterious signaling cascades that cause apoptosis, excitotoxicity, oxidative stress, and inflammatory responses [27]. The recovery phase of IS, in turn, involves physiological activities such as ischemia–reperfusion and angiogenesis, which largely influence the prognosis of the condition. By approaching the pathophysiological basis of IS through various body fluids with multiomics, including transcriptomics, genomics, proteomics, and metabolomics, we can detect molecular changes in body fluids to provide a great pool of information for the study of ischemic stroke.

### Blood

From a hemodynamic perspective, IS involves complex changes at multiple levels. Blood is an important medium to transport oxygen and nutrients throughout the body, and during IS, the blood undergoes a series of reactions and changes that are closely related to the onset, progression, and prognosis of the disease. First, from the transcriptomic level, IS leads to changes in the transcript levels of genes in the blood associated with inflammatory responses, coagulation, and vascular injury. Alterations in these genes, such as the RNA binding protein human antigen R (HuR) and brain endothelial mRNA, may inhibit or exacerbate inflammatory responses, promote thrombosis, or aggravate vascular injury [28, 29], and exacerbate ischemic damage to brain tissue. Second, from the blood proteomic level, it is also significantly altered. In IS, the expression levels of a number of proteins associated with coagulation, inflammation, and neuroprotection are altered. For example, an increase in coagulation-related proteins, such as fibrinogen, may lead to clot formation [30], whereas changes in inflammation-associated proteins, such as fibroblast growth factor 21 (FGF21) and macrophage inflammatory protein-1 alpha (MIP-1α), may be protective of the blood–brain barrier in the event of IS, or induce atrial fibrillation, which exacerbates inflammatory responses in brain tissue, as symptoms brought by condition [31, 32]. Meanwhile, the reduction of some neuroprotective proteins may make brain tissue more susceptible to ischemic injury; e.g., sphingosine-1-phosphate is involved in the regulation of endogenous cerebral vasoprotective signaling, which is expected to be a therapeutic target for IS [33]. Third, from the metabolomic level, IS affects the levels of metabolites in the blood. Because brain tissue has an extremely high energy demand, ischemia leads to changes in energy metabolism pathways. As a result, blood metabolites such as lactate and pyruvate are altered.

All components of blood are relevant for analysis and play an important role in the onset and development of IS. For example, increases in growth factors and cytokines associated with inflammatory responses are strongly associated with chronic fatigue, a common symptom of stroke [34]. These changes may capture the magnitude of the inflammatory response of brain tissue during IS. At the same time, serum metabolites, such as lipids and amino acids, may undergo changes that strongly correlate with the energy metabolism and oxidative stress state of IS.

Plasma consists mainly of water, electrolytes, proteins, lipids, and hormones. In IS, plasma coagulation factors and platelets are likely to be altered and involved in the formation and development of blood clots. In addition, plasma lipoprotein metabolism, which is associated with IS risk and prognosis, may also be affected. From a transcriptomic perspective, RNA expression patterns in serum and plasma, such as lncRNA and circRNA, may also be altered during IS. These RNAs may be derived from damaged brain tissue or other cell types, reflecting cellular damage and stress responses during IS.

### Cerebrospinal fluid (CSF)

CSF, as a body fluid that directly reflects the condition of the central nervous system, is involved in several key aspects of the pathophysiological basis of IS. CSF, as a colorless and transparent fluid present in the cranial and spinal cavities, with the main components of water, protein, glucose, and some cells, plays a crucial role in maintaining the balance of the internal environment of the cerebrospinal cord. During the pathology of IS, the ischemic state of the brain initially triggers an energy imbalance that leads to impaired brain cell function. As the disease progresses, mechanisms such as excitatory amino acid toxicity and oxidative stress further exacerbate brain cell damage and trigger a neuroinflammatory response. These pathological changes lead to neuronal cell death, triggering a cascade of proteomic and metabolic changes in the CSF. The role of CSF in this process cannot be ignored. First, CSF provides essential nutrients to brain and spinal cord cells, ensuring that these cells receive adequate nutritional support in an ischemic state. Second, CSF can remove metabolic products and inflammatory exudates, which can help maintain the stability of the internal environment of the cerebrospinal cord and reduce the damage caused by IS. In addition, CSF has the ability to absorb shock and dissipate pressure, thereby protecting the brain from traumatic injury. Thus, CSF can be a direct reflection of the CNS, reflecting the biochemical response of the brain when IS occurs [35].

### Urine

Urine usually does not directly reflect the pathophysiological changes of IS because it is mainly produced by the kidneys, which filter the blood, mainly removing metabolic wastes and excess water from the body and maintaining the stability of the body’s internal environment. However, IS or its course of treatment may indirectly affect the composition of urine. For example, during the acute or recovery phase of an IS, patients may need to receive medications such as the diuretic Mannitol for neurologic brain edema [35]. These medications may affect urine composition and volume. In addition, if an IS results in kidney dysfunction, it may also affect urine production and composition. Nonetheless, urinalysis remains an important diagnostic tool in medicine to help physicians understand a patient’s overall health, including kidney function and metabolic status. For example, changes in protein, glucose, ketone bodies, and other components of urine may signal problems such as kidney disease or metabolic disorders.

### Saliva

Saliva, as a direct reflection of the oral environment, has a rich and diverse composition, including electrolytes, proteins, enzymes, and microorganisms. In the case of IS, the inflammation-related factors, metabolites, or hormones in saliva may change, and they may indirectly reflect the systemic inflammatory response or metabolic abnormalities triggered by IS. By in-depth analysis of these changes in saliva, we can understand more comprehensively the effects of IS on the systemic physiological status of patients.

### Aqueous humor

Changes in aqueous humor, an important fluid in the eye, are likely to indirectly reflect states such as ischemia or edema in the eye during IS. Any changes in the composition or abnormalities in the kinetics of atrial fluid may be potentially useful markers in the pathophysiological process of IS. By investigating the changes in atrial fluid, we may gain a better understanding of the relationship between IS and ocular status.

### Tears

The tear gland functionality is coordinated in a holistic way being, therefore, affected by systemic processes reflected in the tear composition. Consequently, alterations in the tear composition are relevant for IS risks, disease development, clinical onset, and individual outcomes. Combined with the patient-friendly non-invasive character of the tear fluid sampling, diagnostics utilizing basal tears is expected to play a central role in the 3PM-guided IS management [11].

## Targeting individual body fluids for multiomic analyses in IS

Based on the association degree of those different body fluids and the pathological mechanisms of IS, and the body fluid multiomics studies on IS in recent years, we have compiled the following body fluids as the targets for multiomics analysis, from which we found the corresponding biomarkers to further understand the pathology and clinical management of IS. In this part, we will try to demonstrate the feasibility and credibility of our two hypotheses (hypotheses A and B)**,** making body fluid an instrumental tool for 3PM research of IS and contributing to the next development of stroke healthcare as a public health subject.

### Blood

Different types of blood and components undergo significant changes during the course of IS. Exploring the relationship between blood and its molecular changes and the progression of IS is an important means to uncover biomarkers of IS. Blood biomarkers will help in early diagnosis, disease detection, and prognosis maintenance in IS. Blood is an important target for multiomics analysis of IS. Here, we will describe the discovery of potential biomarkers in whole blood, peripheral blood, and venous blood, as well as serum and plasma, respectively, to demonstrate the feasibility and credibility of our two working hypotheses.

#### Whole blood

In multiomics studies of IS, whole blood is an important targeted body fluid used to discover biomarkers for predicting, diagnosing, treating, and helping the prognosis of IS. There are abundant materials contained in whole blood, not only biomolecules such as proteins and metabolites but also cells from which genes can be extracted in cell nuclears with conditions for sequencing and other materials in akaryotes, and all of these have the potential for the discovery of IS-associated biomarkers.

In primary care, there are many people proposing the 3PM value for IS. For example, RNA expression profiles in the blood can indicate whether a patient has an arterial, cardiogenic embolic, and lacunar etiology and, along with other clinical signs, can help identify and diagnose cryptogenic stroke [36] or elucidate differences in inflammatory responses between stroke subtypes [37]. Therefore, many RNAs are regarded as independent factors and predictors of the risk levels of IS for prevention. Similarly, there are also researchers reporting that the methylated MTHFR significantly increases the risk of IS susceptibility, which provides concrete indicators for IS prevention [38]. Moreover, some metabolites examined in the blood are reported to contribute to stroke recurrence prevention. Decanoylcarnitine and octanoylcarnitine have also emerged as de novo biomarkers for the diagnosis of cardiogenic stroke and the prediction of stroke recurrence [39], which is believed to have benefits from primary care instead of a higher cost in recurrence, while also being functional in secondary care of cardiovascular diseases leading to stroke. Compared with the outbreak of IS or negative prognosis, the biomarkers, part of the IS 3PM research efforts and found in whole blood, are supposed to effectively reduce the economic burdens of IS healthcare, which shows the main ideas of our working hypothesis A.

The whole blood is a frequently used body fluid in clinical practice, as we all know. This is an easy-to-extract fluid with abundant components changing during IS progression and worth for 3PM research. For example, blood-extractable metabolites, such as arginine and acetyl-carnitines, have been shown to be strongly related to stroke severity [40]; it has also been shown that some proteins in the blood can be used as targets for drug therapy or determining prognosis; for example, SCARA5 can be used as a target biomarker for the treatment of cardiogenic embolism and the prevention of subarachnoid hemorrhage, with no evidence of undesirable side effects found [41]; and markers of inflammatory responses, atherosclerosis formation, and the oxidative stress have emerged as the latest promising improvement in the prognosis of IS [42].

Biomarkers extracted from whole blood are reported to make a difference in predictive, preventive healthcare and accurate diagnosis and treatments during pathological progression of stroke. Therefore, the whole blood measures up to the standards to be an effective tool for IS 3PM research, supporting the working hypothesis B**.** Physicians should make full use of these indicators to provide patients with more accurate and personalized treatment plans.

#### Peripheral and venous blood

Peripheral and venous blood indices can also be used as target fluids for IS multiomics studies, and both blood samples can provide rich biomarker information, which can help us to understand more deeply the pathogenesis, pathological process, and disease progression of IS. MMP-2 methylation levels in peripheral blood DNA have been shown to be significantly lower in patients with small-vessel stroke [43], especially in men, than in controls [44], possibly related to the possible role of MMP-2 in disruption of the blood–brain barrier due to insufficient perfusion of brain tissue [45]. Another study examined methylation levels of the miR-223 promoter region and miR-223 in the peripheral blood leukocyte genomic DNA and showed a strong correlation between hypomethylation of the miR-223 promoter and atherosclerotic cerebral infarction, leading to the hypothesis of using MIR-223 in DNA as a potential predictive biomarker for atherosclerosis and as a potential predictive biomarker for cerebral infarction [46]. The methylation level of AHCY DNA extracted from peripheral venous blood was shown to correlate significantly with the degree of risk of IS stroke, and it was further hypothesized that the methylation pattern of this gene was able to be regarded as a diagnostic biomarker for IS [47]. These researches mentioned above are all classic examples that venous and peripheral blood are important in the multiomics study of body fluids in IS and are a valuable resource for gaining an understanding of the pathogenesis of IS, finding new treatments, and assessing prognosis. We strongly believe there are still many other findings and biomarkers still waiting to be discovered to reveal more values for these two kinds of blood, while we accept these examples to support the feasibility and credibility of our working hypothesis B.

#### Serum and plasma

Blood is precipitated or centrifuged to obtain serum or plasma. Serum and plasma, as important components of blood, are of great research importance in IS multiomics studies. Compared with whole blood, serum and plasma are free of blood cells, and contain a large number of biological macromolecules, such as proteins, metabolic products, hormones, and nucleic acids, which play a key role in the pathogenesis, pathological process, and disease progress of IS.

In risk prevention, some biomarkers are reported to be associated with a lower risk of occurrence of IS and potentially contribute to its primary care. For example, there was a causal relationship between lower serum MMP-12 levels and risk of IS, between lower serum MMP-1 and MMP-12 levels and risk of large-artery stroke, and between higher serum MMP-8 levels and risk of lacunar stroke, which can be monitored as indicators for the prediction of susceptible population and targets for medical intervention [48]. Similarly, elevated prekallikrein, MMP-12, and SWAP70 are significantly associated with reduced IS risk [49], and linoleic acid was published to provide a basis for dietary prevention of IS and other cardiovascular diseases [50], which revealed the value for both primary and secondary care. These researches provide the molecular conditions for IS 3PM patterns healthcare, which potentially become accurate, sensitive, and cost-effective markers for primary and secondary care of IS in the future, and reveal the value of working hypothesis A that we put forward.

Moreover, there are tens of hundreds of biomarkers discovered effective for IS 3PM research, no matter for pathological mechanism, clinical practice, and improvement of 3PM patterns. For example, the plasma antimicrobial protein REG3A, which is involved in the validation and cell survival in stroke, has been hypothesized to be a potential biomarker with a prognostic function in predicting stroke within hours [51]; and the plasma candidate differential microbiota-derived metabolite phenylacetylglutamine (PAGln), the levels of which were shown to be associated with IS, especially moderately severe symptomatic-grade short-term adverse outcomes in targeted metabolomics analyses of IS symptoms are highly correlated and are potential research targets for IS diagnosis and prediction of risk level and prognosis [52]. When using serum as a sample, ones detected and compared the differences in serum protein levels between acute-phase strokes and controls with dynamic LC–MS mode, which identified ten proteins with significant differences (including apolipoprotein A-I, fibronectin, thymidine phosphorylase-2, ficolin-2, and beta-Ala-His-dipeptidase) as a potential biomarker and new pharmaceutical compound for the diagnostic differentiation of AIS through further clinical testing [30]; serum-derived DNA molecules ABCG1 and APOE, DNA methylation at the cg 02494239 locus of the former correlates with IS and carotid intima-media thickness (cIMT), while hypermethylation of the latter correlates with ankle-brachial index (ABI), indicating that epigenetic alterations of ABCG1 and APOE could play a role in the pathogenesis of cardiovascular disease, including IS [53].

Serum and plasma are two body fluids that carry a large amount of biomolecules and are often used as research samples and carriers in multiomics studies of IS. In clinical practice, both serum and plasma are often used as assays to aid in the differential diagnosis of disease types and to determine disease progression. Plasma and serum markers are of great importance in multiomics studies of IS and form a strong scientific basis for understanding the pathogenesis of IS, searching for new therapeutic approaches, and assessing the prognosis of IS. There are still many other evidences supporting that plasma and serum are essential fluids to be sampled in the medicine field. All in all, we’ve definitely proved that these two components of blood with blood itself are what our working hypothesis B is supposed to be worthful and creditable.

### Cerebrospinal fluid (CSF)

CSF is of extremely critical importance in multiomics studies of body fluids in IS. Unlike blood, CSF receives degradation products that directly reflect the physiochemical procedures of cerebral tissue, and is therefore an excellent fluid for the early assessment of neurological disorders [54]. CSF acts as a solvent for biomarkers in neural tissues [55]. Several human studies have found that the protein S100B in CSF correlates with the degree of risk of IS and prognostic outcome (as measured by the Improved Rankin Scale) [55], suggesting that S100B is a candidate biomarker for diagnosing and predicting the prognosis of IS; CSF is also enriched in free fatty acids, the levels of which have been reported to correlate with hospital discharge NIHSS and observed stroke volume per beat [56] and have been shown in a 119-person study to help discriminate cardiogenic embolism from non-cardiogenic embolic IS [57], making it a target for stroke diagnosis and prognostic prediction. CSF is the fluid that surrounds the brain and spinal cord and directly reflects the pathophysiologic state of the central nervous system. Therefore, it offers a unique perspective for the multiomics study of body fluids in IS and is an important target fluid for IS research, which supports our working hypothesis B. However, CSF collection usually requires invasive procedures such as lumbar puncture, which increases patient pain and risk of infection. The difficulty and risk of CSF collection are issues that cannot be ignored, which is the reason why CSF is difficult to assess as fluid for everyday healthcare of IS.

### Urine

In the multiomics study of body fluids in IS, urine is of great research interest as a non-invasive and easily accessible body fluid sample. The biochemical components in urine can reflect the metabolic state of the body, renal function, and possible pathological changes, and therefore, it is an important tool in the study of IS. Reduced levels of citrate and glycine in urine, as detected by 1H-NMR spectroscopy, are changes that are strongly associated with folate deficiency and hyperhomocysteinemia [58], which are considered independent risk factors for stroke [59, 60]. This research revealed that IS can be prevented by monitoring levels of metabolites in urine, keeping the disease closely associated with stroke away, which explains our working hypothesis A. Since urine is a kind of non-invasive and easy-to-get body fluid, a survey of some metabolites of urine is worth being applied in IS prevention and prediction and may provide conditions for considerable cost-effective benefits in the healthcare of individuals.

As reported, citrate and glycine are associated with folate deficiency and hyperhomocysteinemia. This finding indicates the preventable risk factors of IS in primary care, but as a disease related to stroke, it can also be regarded as evidence for IS secondary care. Therefore, biomarkers discovered in urine are worth discussing, many of which contribute to both primary and secondary care. In secondary care, a study comprised 194 DEPs, which indicated that the six urinary proteins ACP2, PLD3, HLA-C, GGH, CALML3, and IL2RB had high sensitivity and specificity in differentiating between asymptomatic and symptomatic carotid artery stenosis (CAS), and showed potential value for the diagnosis of further CAS-induced strokes, and could be used as potential CAS stroke diagnostic biomarkers and targets [61]. Another study showed that five of the ten urinary metabolites (glycine median, sarcosine median, methionine median, aminoadipic acid median, and oxaloacetate median) associated with stroke in the acute or chronic phase have a correlation with amino acid metabolism relationship and can therefore be considered related to changes in oxidative stress in stroke and participate in the pathologic mechanisms of IS [62]. Molecular patterns demonstrated in urine are revealed to be beneficial for both risk factor prevention and pathology progression, the application of which probably be effective for predictive diagnosis and disease healthcare, what we say hypothesis B. By analyzing the biochemical components in urine, we can gain insight into the pathogenesis, pathological processes, and disease progression of IS, providing new strategies and tools for early diagnosis, treatment, and prognostic assessment of the disease.

### Saliva

As a readily accessible and non-invasive form of human body fluid, saliva represents a rich source of samples useful for multiomics studies of body fluids. It has a rich variety of biomarkers, including proteins, nucleic acids, hormones, enzymes, and other biomolecules, which are potentially valuable in the diagnosis, treatment, and prevention of diseases. Saliva is an important fluid for the study of IS 3PM. For example, one study detected salivary cortisol as an independent risk element for mild cognitive impedance (MCI) after CIS. In this case, researchers put forward that salivary cortisol levels can be used as predictive biomarkers for MCI, as primary care for preventable complication of IS [63]. Similarly, it has also been shown that salivary xanthine oxidase takes part in biochemical reactions during the occurrence of ischemia–reperfusion; its activity is used with very high specificity and sensitivity as a biomarker for the diagnosis of ischemic and hemorrhagic stroke, and its functional status [64] also plays an accuracy role in distinguishing mild-to-moderate cognitive decline. Saliva can also reflect the level of stroke risk by reacting to oral infections in patients with IS [65]. These findings collectively support the feasibility and credibility of our working hypothesis A. When compared with blood and other body fluids, saliva is simply and conveniently collected and doesn’t require special medical equipment or specialist training, which greatly lowers the cost and threshold of research. Saliva contains many biomarkers that play a role in reflecting both the clinical phenomenon and complication of IS and may contribute to lower IS prognosis healthcare cost and effectively prevent the occurrence of other serious complications.

In addition, with the continuous development and improvement of histologic technology, saliva will be more and more widely used in multiomics studies of body fluids. First, in the process of exploring biomarkers of inflammation, saliva is gradually showing unique potential. For example, circRNAs, as a new type of biomarker, play an important role in signal transduction and the inflammatory response, and the impact of circRNAs on the progression of many long-term diseases, such as neurovascular diseases, cancer, inflammation, and diabetes, which is worth exploring further [66, 67]. Second, for neurological disorders, saliva is also an important sample for reacting to disease course and predicting diagnosis. In addition to the aforementioned oral infection profile that reflects the degree of stroke risk, it has been shown that salivary Aβ42, tau proteins, certain metabolites, and oral microbiota have predictive biomarker value for the diagnosis of Alzheimer’s disease (AD) [68]. Salivary BDNF gene methylation is also a test for borderline personality disorder (BPD) in other neurological disorders [69].

In summary, saliva’s value as a body fluid for brain diseases is supposed to be focused further. All these mentioned researches reveal that saliva is an instrumental tool that can be applied in predictive diagnosis, differentiation, prognosis, and primary and secondary care of IS and other brain and neuro diseases, supporting our working hypothesis B**.**

### Aqueous humor

The aqueous humor is a clear, lucid fluid that fills the anterior and posterior regions of the eye and is manufactured by the epithelial cells of the ciliary processes. In a setting of IS, aqueous humor, although not directly involved in the blood supply to the brain or in pathological processes, may reflect indirectly certain systemic changes associated with IS. For example, cGLIS3 in aqueous humors was found to increase significantly in IS compared to controls. It has been proposed that the retina is a brain window and cGLIS3 in aqueous humors is a regulator and predictive diagnostic marker of brain neurodegeneration from a circRNA perspective [70]. This finding may provide one with clues about the pathogenesis of IS, disease progression, and treatment efficacy.

The aqueous humor is comparatively easy to acquire and be gathered and examined during ophthalmic surgeries. As a consequence, there are many scientific research findings on the prediction, diagnosis, and personalized therapies of diseases through aqueous biomarkers, predominantly eye-associated diseases. Among them, the acquisition and analysis of aqueous humor are of great clinical importance in human diabetes-induced ocular and systemic diseases, which are demonstrated to be related to IS and regarded as secondary care [21]. For example, in diabetes-induced nonvalue-added retinopathy, aqueous humor can be utilized for pre-clinical prediction, diagnosis, and determination of the disease stage of diabetes-induced retinopathy by metabolism and proteomics through changes in a number of protein molecules, such as interleukin, apolipoprotein A-I, and brain-specific angiogenesis inhibitory factor 1-associated protein 2 (BAIAP 2), without ocular surgery [71, 72]; detectable differential proteins in aqueous humors can be used to infer the pathogenesis and course of diabetic nephropathy [73]. These findings support our working hypothesis B and show the value of aqueous humors for IS secondary care application. Overall, the detection of aqueous humor offers new opportunities for liquid biopsy in patients, and provides new perspectives to study pathological mechanisms and provide 3PM biomarkers in addition to directly reflecting ocular diseases, but also in other systemic diseases, such as diabetes mellitus, neoplasms, and stroke.

### Tear fluid

Among several types of tears, specifically, basal tears are considered the most valuable source of information relevant for predictive and companion diagnostics [74–77]. Basal tears, readily available for patient-friendly non-invasive sampling, contain cell-free DNA, amino acids, proteins, metabolites, hormones, lipids, and other biomolecules collectively reflecting systemic effects which are relevant for IS risks, development, and clinically manifested pathology. To this end, quantitative PCR analysis of cf-mtDNA demonstrated great clinical utility for detecting mitochondrial burnout by extremely low levels of mitophagy measured in the tear fluid that is highly relevant for both IS and sudden heart arrest (the know-how of “3PMedicon GmbH” performing internationally validated tests (3PMedicon your risk reducer; https://www.3pmedicon.com/en/scientific-evidence/compromised-mitochondrial-health)). This test is routinely applied for health risk assessment in primary care provided to individuals with FSP frequently demonstrating silent lacunar ischemic lesions in the brain (asymptomatic IS) [20] which supports the feasibility and credibility of our working hypothesis A. Since impaired mitochondrial health and homeostasis strongly contribute to cerebral ischemia–reperfusion damage, tear fluid analysis focused on disease-specific mitophagy patterns is of high clinical relevance for both predictive diagnostics and advanced IS treatments and may have enormous cost-effective benefits to the health of affected individuals and healthcare at large [11, 20].

**Fig. 3:**
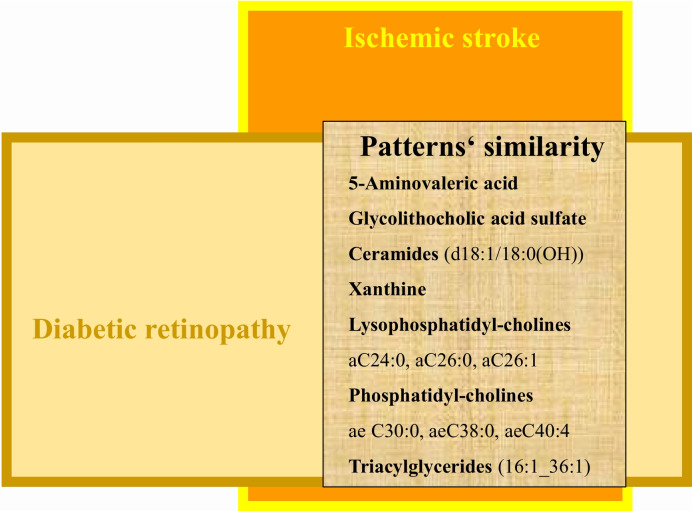
Tear fluid as the source of molecular patterns for health risk assessment and predictive and companion diagnostics in secondary care; patterns’ similarity demonstrated for PDR patients with clinically manifested IS; the image is adapted from [11]

In secondary care, DM patients are generally at increased risk of IS, but by far, not every DM patient is predisposed to the cerebral infarction. In order to avoid both under-treatment and over-treatment, an accurate patient stratification is essential for individualized IS prediction in the DM patient cohort. A fascinating achievement was recently presented by the EPMA international expert group who was able to demonstrate metabolic patterns in the tear fluid shared by both IS patients and DM ones who developed diabetic retinopathy [11, 22]. To this end, PDR is an early indicator of cascading pathologies secondary to DM including IS [17] (see Fig. 3). Among metabolites, 13 molecular clusters relevant specifically for cardioembolic IS have been identified and quantified in basal tears [11]. All the identified molecular clusters are per evidence involved in key pathomechanisms that underlay IS risks, clinical manifestation, and poor individual outcomes such as imbalanced oxidative stress and stress-driven anxiety, altered mitophagy patterns and sleep–wake behavior, mitochondrial respiratory chain dysfunction, inflammaging, vascular stiffness, and remodeling, among others [11]. These findings collectively support the feasibility and credibility of our working hypothesis B, making tear fluid a non-invasive, patient-friendly, attractive source of information for health risk assessment and predictive and companion diagnostics in advanced IS management.

## Techniques to analyze multiomic patterns

### Genomics

#### Genomic techniques

Genomics provides guidance for the diagnosis, treatment, prognosis, and individualization of disease through the study of the structure, function, and interconnections of genes. In 2001, the scientific community proclaimed the ultimate success of the Human Genome Sequencing [78], after which the human population reached a deeper stage in its understanding of genetics and variation. Genome-wide association studies (GWAS) analyze associations between DNA variants and traits based on the principle of linkage disequilibrium (LD) in population genetics. Genome-wide association studies typically require large numbers of samples to identify reproducible genome-wide significant associations. In addition, the number, effect, and LD between observed genotyped DNA variants and unknown causal variants segregated in a population all influence the strength of the genetic connection to a particular condition or trait [79]. Clearly, GWAS is influenced by multiple factors and cannot identify all genetic determinants of complex diseases such as IS [80]. However, GWAS has significant advantages in capturing racial variation in complex traits, and its data generation and analysis are relatively simple. The utilization of public databases has facilitated new discoveries and made GWAS an indispensable player in genomics research [80].

Genes mediate human phenotype and structure and have corresponding reflections and forecasts in the disease as well. Gene genomics technology has a central role in the fluid-based multiomics study of the IS, and as the starting point of the DNA extraction technology, it assures the integrity and purity of the DNA in the samples. The PCR technique subsequently plays a key function in the fields of gene and genotype cloning with its efficiency and simplicity. Gene sequencing technology, on the other hand, is a central technology for the precise sequencing of DNA, which sheds light on the structure and function of an organizer’s genome and has provided important insights into the mechanisms underlying the genesis of IS. Next-generation sequencing technology provides guidance for individualized medicine by detailed analysis of exome and genome data to analyze relationships between genetic mutations and phenotype of patients [81]. Whole-exome sequencing determines disease-causing mutations through the analyzation of protein-encoding regions in the genome, which can guide clinical disease diagnosis, assessment of disease risk, and precision medicine. However, an evident disadvantage of whole-exome sequencing is that it only detects sequences in the exonic regions of the genome, resulting in lower cost and labor intensity, but it cannot analyze the influence of DNA sequences outside of the epitope on the phenotype. Hence, whole genome sequencing has been a solution. By sequencing both the protein coding and non-protein coding areas of the genome, genetic variation can be tested throughout the genome. Whole genome sequencing has more advantages in testing for migrations within exon regions as compared to whole exon sequencing [82]. In addition, gene editing technologies such as CRISPR/Cas9 have offered new potential for precise therapeutic treatments. These genomic technologies together form a solid basis for IS humor multiomics studies, offering strong support for our in-depth understanding of the underlying molecular mechanisms of the condition and for the discovery of new approaches to treatment.

#### Epigenomic techniques

Epigenomics is the study of modified DNA without sequence changes, with differences in DNA methylation levels being the main focus in the progression of IS. Methylation is one of the first forms of epigenetic modification of genes to be identified and plays a key role in the control of gene expression. The main methods for DNA methylation detection are sequencing technologies based on the principle of bisulfite conversion, such as bisulfite sequencing, pyrosequencing, whole genome bisulfite sequencing (WGBS), reduced representative bisulfite (RRBS), more recently developed single-molecule, real-time bisulfite sequencing (SMRBS), and bisulfite sequencing (SMRT-BS) [83]. Other common techniques that do not utilize sodium bisulfite to convert cytosine residues to uracil/thymine include methylated DNA immunoprecipitation (MeDIP) and methylated DNA immunoprecipitation sequencing (MeDIP-sequq), which combine immunoprecipitation with sequencing techniques. The limitation of sequencing techniques based on sodium bisulfite treatment is that it reduces the accuracy of sequence alignment. Ion semiconductor sequencing is a simple and efficient method with similar accuracy to Sanger sequencing but faster; however, it cannot distinguish between rare 5hmC and 5mC modifications [84, 85]. Compared to WGBS and RRBS, RRBS increases the coverage of CpG islands by enzymatic cleavage while reducing the genomic scope of the evaluation. Compared to WGBS and RRBS, MeDIP-seq expanded genomic coverage without reducing sequence alignment; however, it was limited by the inability to analyze methylation at single-base resolution and was more challenging to perform [86].

Epigenome-wide association studies (EWAS), inspired by genome-wide association studies, focus on identifying different methylation sites by analyzing the association between DNA methylation status and disease-related traits. With the development of microarray technology and various high-throughput sequencing technologies, more and more gene loci are covered by the whole epigenome, which brings new challenges to data analysis and processing. On the other hand, unlike GWAS, natural variation affects DNA methylation characteristics, which may bring some errors to the evaluation of results. In addition, the integrated use of different omics technologies can help to provide more comprehensive information; e.g., proteomic analysis and integration of RNA sequencing data with transcriptional profiling can be used to identify the downstream target of a certain RNA, which can be used as a biomarker and further elucidate the molecular mechanism of the disease.

### Transcriptomics

Transcriptomics analyzes the types of ribonucleic acid (RNA) produced in cells, such as mRNA, rRNA, tRNA, and other non-coding RNAs, providing information on numerous RNA expression profiles. Transcriptomics techniques are playing an indispensable part in the multiomics study of body fluids in IS. Transcriptomics is a subject that examines the state of gene transcription and the laws that govern transcriptional regulation in cells, which concerns the transcriptional progression from DNA to RNA as in the processing of RNA and its ultimately being expressed as proteins. The bioinformatic analytic techniques, such as microarray analysis technology, is used to measure transcript abundance by hybridization with fluorescently labeled complementary probes, which consist of short nucleotide oligomers attached to a solid surface [87, 88], helping us to mine valuable information, such as differentially expressed genes and transcription factor binding sites, from massive transcriptome data, which can provide important research on the pathogenesis of IS and drug therapeutic target discovery. However, the disadvantages of microarray technology are the need for prior knowledge of the reference transcriptome required for probing, and the application of microarray technology cannot detect additional or novel variants [89].

Compared to microarray technology, RNA-Seq technology is the latest transcriptomics research technology. As a core tool for transcriptomics research, high-throughput sequencing technology, RNA-Seq, is capable of acquiring transcript information, both mRNA and non-coding RNA, in a thorough and efficient manner, so as to reveal the pattern of gene expression and control mechanism under varying conditions. On the basis of the high-throughput seeding principle, RNA-Seq determines the richness of transcripts by ordering and counting the transmitted cDNAs. Although sample preparation and data analysis are more complex compared to microarray technology [90], RNA-Seq can detect SNPs and splice variants [91]. Due to the significant advantages of RNA-Seq in terms of throughput, accuracy, and read length, it has become a mainstream technology for transcriptomics [92].

In addition, single-cell genome-wide sequencing of the transcriptome can be carried out using the microfluidics technology [93]. Exon arrays allow exon-level data to be available for studying the consequential splicing events [94]. In conclusion, integrating emerging technologies together with key techniques of transcriptomics has helped to explore the link between gene expression and cellular reactions to disease, and transcriptomics technologies have a broad perspective of application and substantial scientific worth in humoral multiomics studies of IS.

### Proteomics

Proteomics is a massive, high-throughput study of proteins and the overall protein system [95], and its analysis of protein architecture and features provides a scientifically accurate way to explore the pathological and molecular mechanisms of diseases. Proteomics technology also takes on a pivotal role within the multiomics study of body fluids in IS. Proteomics-related analytical techniques have been progressively applied to clinical aspects, which adds a highly precise and sensitive pathway for molecular studies of stroke. Among them, the combination of two-dimensional gel electrophoresis (2-DE) and mass spectrometry (MS) techniques can accurately isolate and identify proteins in body fluids and unveil their association with IS [96]. Nowadays, the integration of 2-DE and MS has become a comparatively well-established technique in proteomics. Moreover, other hybrid technologies in proteomics are also developed, including liquid chromatography (LC)-MS, gel electrophoresis-LC–MS, capillary electrochromatography-MS, and other chromatographic techniques coupled with MS, for the accurate analysis of proteins [97–99].

In addition, quantitative proteomics technology, including isotope tagging for relative and absolute quantification (iTRAQ), tandem mass targets (TMT), SILAC, and label-free, provides a powerful tool for protein quantification studies with its high-throughput and high-precision features; protein interaction network analysis is helpful for ones to gain a deeper understanding of the interactions between proteins and their functionality in disease development; and the modification site analysis in protein further reveals the role of protein modification in IS. On the basis of a number of techniques, the scientific community has developed proteome-wide microarrays to evaluate immune responses, screen biomarkers, and apply them in clinical practice [100]. These technical approaches complement each other and together form a framework for the proteomics study of body fluids in IS.

### Metabolomics

Metabolomics reflects multifactorial, dynamic, and metabolic reactions to both pathological and physiological perturbations in vital systems [101], and qualitative or quantitative analysis of metabolites in the disease process provides insights into the etiopathogenesis and pathological process of the disease. Among the multiomics studies of body fluids in IS, metabolomics is also developing rapidly, mainly including nuclear magnetic resonance (NMR)-based metabolomics and MS-based metabolomics. NMR, with its advantages of non-destructive and quantitative, provides strong support for resolving small molecular metabolites in body fluids. LC–MS and gas chromatography-MS (GC–MS) techniques, with their high sensitivity and resolution, assist ones to understand the change of complex metabolites in-depth, with their high solution resolution and accuracy in the discovery of new metabolite markers and the revelation of their disease effects.

Metabolomics is the novelty of recent years in histology research. The techniques of metabolomics are more based on the basis of other histologies and are very connected with the development of other histologies. In combination with genomics, transcriptomics, proteomics, and other multiomics, metabolomics studies the molecular mechanism of disease from the perspective of metabolite variation. When used for screening biomarkers, metabolomics is more frequently applied in combination with statistical methods such as principal components analysis and least squares regression. Together, these techniques complement one another and constitute a powerful technical system for multiomics study of body fluids in IS, providing an important scientific basis for revealing the metabolic process of the disease and promoting the progress of diagnosis and treatment.

### Statistical methods

In addition to the above-mentioned multiomics analysis techniques, many statistical methods are used for body fluid multiomics in IS, such as Mendelian analysis. The Mendelian analysis is a statistical analysis method based on the genetic principles, which is used to examine the impact of hereditary factors on IS. Using Mendelian analysis, researchers can assess the association between specific genes or genotypes and the risk of IS, which in turn sheds light on the role of genetic variations in the development of the disease. In parallel with Mendelian analysis, there are other statistical methods that play an active role in the multiomics analysis of body fluids in IS. As an example, correlation analysis can help to identify associations between different histological data (e.g., genome, transcriptome, proteome, and metabolome) in body fluids, revealing their interactions in IS. Cluster analysis can categorize samples or molecules with similar characteristics, which can help to discover new disease subtypes and potential therapeutic targets. In addition, methods such as principal component analysis and partial least squares regression can also be used for the reduction of data dimensionality and feature extraction, increasing the analysis’ efficiency and accuracy. These statistical methods are able to sift valuable information from massive data collected, through which we discover new potential biomarker and provide novel condition for early diagnosis and assessment of the prognosis of diseases.

## Multiomic patterns in body fluids are instrumental for predictive approach, preventive intervention, and companion diagnostics

In our in-depth exploration of IS research, ones have integrated multiomics research methods, including genomics, transcriptomics, proteomics, and metabolomics, to completely unravel the pathogenesis of IS and seek potential therapeutic targets. Genomics studies have identified genetic variants and hereditary factors associated with IS risk, providing important clues for understanding the genetic background of stroke. Meanwhile, transcriptomics studies have revealed changes in gene expression during IS, helping ones to gain insight into the molecular mechanisms of IS. Proteomics studies have focused on the changes in protein expression and function caused by ISE and have identified specific protein markers associated with IS, providing new possibilities for diagnosis and treatment. Metabolomics studies have revealed changes in metabolites in stroke patients, providing an important basis for understanding abnormalities in metabolic pathways and developing therapeutic strategies. This part introduces the integrated biomarkers distributed multiomics fields separately, mainly explaining the concrete components and examples of hypothesis B in 3PM patterns of IS, and indirectly shows the value of hypothesis A, from which we infer the actual medical and economic effects of the body fluid multiomics attached healthcare of IS. In summary, through the comprehensive use of multiomics approaches, ones have provided new perspectives for the in-depth study of stroke and new ideas and methods for prevention, diagnosis, and treatment. These findings are expected to advance the field of stroke care and improve patient outcomes and quality of life.

### Genomic patterns

#### IS relevant genomic patterns

Genomics has always played an important role in IS multiomics research. Humoral genomics allows the systematic study of changes in genes, transcripts, proteins, and other biomolecules in body fluids to reveal specific biomarkers associated with IS. With the advent of global genome analysis and sequencing technologies, an increasing number of DNA sequence variants have been found to be associated with disease. The study of potential genetic risks can help to further explore the biological processes of the disease and provide new insights for early diagnosis and therapeutic targets. Nowadays, researchers have identified many gene loci and SNPs associated with IS, which were revealed to be potential biomarkers for IS PPPM. Through the typical genomic technique GWAS, researchers found many IS-related SNPs, which affect or belong to some gene sequences. It is reported that sequence variations of some genes are associated with disease pathological process. For example, the sequence variation of HDAC9, which is involved in encoding histone deacetylase 9, was found to be associated with LAA stroke [102]. Similarly, ABO gene variations are associated with large vessel and cardioembolic stroke, while ZFHX3 sequence variations are associated with IS. The involvement of these two genes in the coagulation response and neuronal differentiation influences the pathology of different subtypes of stroke [103–105]. In addition, changes in some gene sequences aid in the diagnosis and treatment of IS. In fact, some researchers have extrapolated clinical values from associations of sequence variations with IS, such as PITX2, BCL3, and PBX3, which were considered potential biomarkers for the diagnosis of IS and LAA stroke [106, 107], and some research showed extrachromosomal circular DNA (eccDNA) can be used as a potential diagnostic marker of LAA stroke [108]. What’s more, with the technique of exome sequencing, PDE4DIP and ACOT4 involved in vivo metabolic reactions such as fatty acid, the variations of which tend to be biomarkers for the prediction, prevention, and treatment of IS [109].

All in all, variations of gene sequences during IS process mean genomic and molecular basis behind the clinical symptoms of diseases. In recent years, people have done a lot of research with high-throughput techniques to find out the pathology and molecular mechanism of IS at a deeper level, for which they discovered many gene sites on any autosomal chromosomes or mitochondrial chromosomes and SNPs involved in pathology reactions or associated with the disease process. Table 1 summarizes the genetic variants associated with IS identified by genomic approaches.

**Table 1:** Typical genomics body fluid biomarkers of IS. *LAA*, large-artery atherosclerotic stroke

SNP	DNA type/chromosomal loci	Gene/affected gene	Approach	Function	Stroke subtype	Application in PPPM	Refs
rs11984041	7p21.1	HDAC9	GWAS	Coding for histone deacetylase 9	LAA stroke	HDAC9 variation is associated with LAA stroke	[102]
rs505922	9q34	ABO	GWAS	Regulation of coagulation	Large-vessel and cardioembolic stroke	ABO gene variants are associated with large-vessel and cardioembolic stroke	[103, 104]
rs7193343-T	16q22	ZFHX3	GWAS	Involved in the differentiation of neurons and skeletal muscle	IS	The sequence variation of ZFHX3 is associated with ischemic stroke	[105]
rs556621	6p21.1	BCL3	GWAS	——	LAA stroke	For the diagnosis of LAA stroke	[106]
rs2200733	4q25	PBX3 PITX2	GWAS	Involved in sinoatrial node development	IS	For the diagnosis of IS	[107]
rs10033464 ——	eccDNA	eccDNA (Chr2:12,724,406–12724784, Chr4:1,867,120–186272046, Chr4:186,271,494–186,271,696, Chr7:116,560,296–116560685, and Chr11:57,611,780–5761192)	High-throughput circle-sequencing technique	Regulation of coagulation and fibrinolysis	LAA stroke	It suggests that extrachromosomal circular DNA (eccDNA) can be used as a potential diagnostic marker of LAA stroke	[108]
rs1778155	1q21.1	PDE4DIP	Exome sequencing	PDE4DIP: interacts with phosphodiesterase 4D	IS	For the prediction, prevention, and treatment of IS	[109]
rs35724886	14q24.3	ACOT4		ACTO4: participating in fatty acid metabolism			

#### IS relevant epigenomic patterns

DNA methylation, the most widely studied epigenetic modification, regulates gene expression and influences biological processes mainly through covalent modifications of cytosine and guanine residues (CpG sites) [110]. Changes in DNA methylation patterns are detected in many diseases, and this close association with clinical phenotypes makes it a potential biomarker for disease exploration. With the development and improvement of detection techniques, altered DNA methylation patterns have been shown to be associated with IS [111, 112]. Whole peripheral blood is the main body fluid sample in IS epigenomics research, with the main technique of pyrosequencing and minority utilization of EWAS, PCR, and array. Some methylated DNAs related to IS were demonstrated to be biomarkers for IS mechanism exploration, predictive diagnosis, prevention, and discovery of new personalized therapy targets, which will be explained below (Table 2).

**Table 2:** Typical epigenomics body fluid biomarkers of IS. *LAA*, large-artery atherosclerotic stroke

gene	Sample	Body fluid	Approach	Function	Findings	Application in PPPM	Refs
MTHFR	IS patients	Whole peripheral blood	Pyrosequencing	Regulates serum folate and vitamin B12 levels	Methylated MTHFR significantly increases the risk of ischemic stroke susceptibility	Providing concrete indicators for IS prevention	[38]
MMP-2	IS patients	Whole peripheral blood	Pyrosequencing	Involved in extracellular matrix remodeling	Low methylation levels of MMP-2 are associated with small-vessel stroke in men	Providing a basis for specific diagnosis and treatment of IS	[44]
AHCY ABCG1	IS patients IS patients	Whole peripheral blood Peripheral blood leukocytes	methylation-specific PCR (qMSP) Pyrosequencing	Sadenosylhomocysteine hydrolase (AHCY) can alleviate the inhibition of Sadenosylhomocysteine hydrolase (SAH) on S-adenosylmethionine (SAM)-dependent transmethylation Involved in lipid metabolism in the body	The high methylation level of AHCY DNA can be used as a diagnostic marker for IS Methylation of cg02494239 in ABCG1 can be used for the diagnosis of ischemic stroke and provide ideas for treatment	Biomarkers that can be used as predictive diagnostics for IS Further exploration of the role of ABCG1 in IS to help stop the course of IS disease	[47, 53]
LINE-I ERα	IS patients IS patients	Peripheral blood leukocytes Whole peripheral blood	Pyrosequencing Pyrosequencing	Retrotransposons Reduced neuronal damage	Lower levels of LINE-I methylation increase the risk of IS in men Women have lower levels of Erα methylation in large-artery and cardioembolic stroke subtypes	Exploring the mechanisms of IS pathology Improvement of personalized treatment mechanism for women with IS	[113, 114]
KCNQ1	Obese IS patients	Peripheral blood leukocytes	Illumina methylation 27 BeadChip array	Potassium voltage-gated potassium channel	KCNQ1 hypermethylation in obese IS patients	Potential biomarkers for predictive diagnosis of IS	[115]
MTRNR2L8	LAA stroke patients	Whole peripheral blood	EWAS	Cell death and apoptosis were inhibited by interaction with Bax protein. Neuroprotective	The evaluation of MTRNR2L8 gene methylation site can guide the diagnosis, treatment, and prognosis of LAA stroke	Its methylation is a diagnostic biomarker and potential therapeutic target for IS	[116]

First, the discovery of DNA methylation level variation contributes to IS pathological mechanism research. For example, LINE-1, whose low methylation has a role in many chronic diseases such as cancer and atherosclerotic diseases and is associated with triggers cell senescence and LDL and HDL metabolism [117, 118], was demonstrated that its lower level of methylation increases the risk of IS in men [113]. Therefore, this gene’s methylation level can not only be a kind of personalized diagnosis and therapy biomarker, but also owns the possibility to explore the concrete pathological mechanism of IS. Second, DNA methylation helps the prevention of IS. Besides the gene LINE-1, there are other genes associated with the risk of IS. For example, gene-methylated MTHFR significantly increases the risk of IS susceptibility, providing the concrete indicators for IS prevention [38]. Third, research on DNA methylation variation makes differences in IS predictive diagnosis and the discovery of new personalized therapy target. It is reported that women have lower levels of Erα methylation in large-artery and cardioembolic stroke subtypes, which didn’t show in the men group [114]. The significantly different levels of gene methylation between different genders revealed IS gender differences in morbidity, which indicated the importance of personalized therapy solutions at a sex level. Similarly, some people found low methylation levels of MMP-2 are associated with small-vessel stroke in men [44], and AHCY gene methylation was significantly higher in IS than in control in both men and women or in different age groups [47]. What’s more, targeting a special population, researchers found KCNQ1 hypermethylation in obese IS patients [115], and for LAA stroke patients, MTRNR2L8 gene’s average number of CpG sites altered significantly [116], which means methylated DNAs are able to be a biomarker for predictive diagnosis or personalized treatments of special patients. Table 2 summarizes the methylation site variations discovered using epigenetic approaches.

### Transcriptomic patterns

Humoral transcriptomics, as a research strategy, is important for the multiomic study of humoral fluids in IS. Transcriptomics broadly studies the collection of all transcriptome products, including mRNA, ncRNA, and rRNA, in a cell under a certain physiological condition, and its research object is specific to the sum of all RNAs that can be transcribed by the cell in a certain functional state. This characteristic enables transcriptomics to deeply explore the mechanism of gene expression regulation and reveal the molecular changes in the process of disease development. Humoral transcriptomics can reveal the dynamic changes in gene expression in vivo during the development of IS by analyzing RNA transcripts in body fluid samples. Through RNA microarray, real-time PCR, and other technologies, researchers sampled body fluids from IS patients as well as controls and then extracted RNA for sequencing and analysis to study the differential effects of IS on human RNA expression, and screened the differential RNAs as candidate biomarkers to further identify diagnostic and prognostic indicators and potential therapeutic targets for IS. In IS transcriptomics research, non-coding RNAs are the main target, including circular RNA (circRNA), long non-coding RNAs, and microRNAs (miRNA).

Loop RNA formed by reverse exon splicing is implicated in transcription, splicing, and chromatin recycling regulation [119]. There is growing evidence that cyclic RNAs are associated with a variety of pathological processes, such as the alleviation of non-alcoholic hepatitis, mediation of drug resistance, and modulation of immunity [120–122]. The study found that circRNAs regulate neuronal development and plasticity [123], and it also heralds the potential of circRNAs as IS biomarkers. In recent years, RNA microarray technology found that circRNAs have high research value in discovering new therapeutic targets for IS and improving prognosis. For example, as for the AIS, which is characterized by an acute condition and poor treatment prognosis, the rate of high expression of circFUNDC1, circPDS5B, and circCDC14A in plasma during the first 7 days of the disease process can serve as potential biomarkers for AIS predictive diagnosis and outcome [124]. Similarly, circRNAs like circSCMH1 in plasma, the low expression of which also can be regarded as the biomarker to evaluate the prognosis of AIS stroke patients, can be used as a therapeutic target to improve the prognosis [125]. For IS cases, researchers discovered that the high expression of circHECTD1, whose coupling reactions provide evidence for translation in IS mechanism, became a new potential therapeutic target [126]. Hsa_circRNA_0003574, which is highly expressed in IS caused by ICAS patients’ whole peripheral blood, was researched as a novel biomarker of ICAS-caused IS and a potential site for IS personalized therapy [127]. What’s more, in circRNAs-related MACO mouse model development and research, some researchers found cGLIS 3, a kind of circRNA extracted from aqueous humor of IS mice, highly expressed during the pathological process of IS, and surmised cGLIS 3 could be regarded as a potential biomarker for the diagnosis of cerebral and retinal nerve co-morbidities in human beings [70].

Long non-coding RNAs (lncRNAs) are transcripts longer than 200 nucleotides that are involved in a variety of biological procedures such as immune response, cell differentiation, and angiogenesis [110, 111, 128, 129]. Significant alterations in lncRNA expression are observed in the serum of IS patients [112], and found that the dysregulated lncRNAs produced after IS were highly homologous to exons [125]. Subsequently, lncRNAs were shown to be involved in the regulation of inflammatory responses, vascular injury, microglia activation, and neuronal apoptosis after IS [130–133]. For example, SNHG15 in peripheral blood and mononuclear cells, which has a role in inhibiting the peripheral inflammatory responses, was revealed to increase during the process of AIS; being a negative regulator of inflammation has the potential to be a new potential therapeutic target for IS [134]. Likewise, another kind of lncRNA, lncRNA-ENST00000568243, extracted from whole peripheral blood, was found highly expressed in IS patients compared to control groups [135]. Researchers revealed it could be regarded as a potential biomarker for the diagnosis of IS.

MicroRNAs (miRNAs), approximately 21–25 nodules in length, are post-transcriptional regulators of gene expressed and are relevant to a wide range of diseases including IS [136]. The study found remarkable variations in the expression profiles of miRNAs in the peripheral blood of patients with IS, showing that stroke has an imprint on the levels of circulating miRNAs [137], and miRNA’s level reflects the course of stroke. Through the RNA-seq technique, researchers found the variations of some miRNA levels of IS patients can help predictively diagnose different types of IS, such as miR-125a-5p, miR-125b-5p, and miR-143-3p in serum, whose high changes were used to identify AIS and TIA [138], and high expression of Hsa-miR-548c-5p in serum was reported to contribute to diagnose AIS [139]. What’s more, research showed miR-424 decreased in the whole peripheral blood of IS mice models. As miR-424 is believed to own the function of inhibiting oxidative stress, the researcher suggested that it can be considered an IS potential therapeutic target in human beings [140, 141].

The body fluid samples for transcriptomics studies are dominated by blood and its components. From extraction of serum, plasma, or whole peripheral, some non-coding RNAs’ variations were demonstrated to be related to the pathological process of IS and considered biomarkers for IS predictive diagnosis, prognosis improvements, and personalized therapy. However, the transcriptomic research also includes values of other body fluids. For example, it has been shown that elevated circRNAs such as cGLIS 3 in the aqueous humor in a mouse model are correlated with degeneration of the cerebral and optic nerves, raising the idea that the retina is a window for the study of brain dysfunction [70]. In recent years, in order to deepen IS transcriptomics research, researchers have established many animal models. For example, in the IS mouse model, it was found that IS mice knocked down with circHECTD1, a circRNA, showed a significant reduction in apparent infarct area, diminished neuronal deficit, and amelioration of glial cell activation in astrocytes in tMCAO mice [126]; other studies have found that delivery of adenovirus targeting SNHG 15 ameliorated stroke-induced immunosuppression in mice, thus inferring new potential therapeutic targets [134]. These studies further confirm the idea of miRNAs, lncRNAs, and circRNAs as predictive diagnostic biomarkers and therapeutic targets for IS. However, despite the confirmation of RNA knockdown and targeting experiments in rodents, there are still significant differences in gene expression between humans and rodents, which may depend on differences in immune responses between humans and rodents and may also be related to differences in the mechanisms of stroke patterns. Nowadays, for human somatic transcriptomics studies with widespread use of microarray technique, total RNA in peripheral whole blood was analyzed, numerous differentially expressed genes were summarized, and the etiology of IS was explored in conjunction with imaging [137], searching for potential diagnostic, predictive biomarkers, and new therapeutic targets for IS. Table 3 summarizes some potential biomarkers identified through transcriptomic approaches.

**Table 3:** Typical transcriptomics body fluid biomarkers of IS. *AIS*, acute ischemic stroke. *ICAS*, intracranial atherosclerotic stenosis. *TIA*, transient ischemic attack. *MACO*, middle cerebral artery occlusion

Biomarkers	RNA type	Approach	Sample	Body fluid	Function	Variations in patients	Application in PPPM	Refs
cGLIS 3 circFUNDC1	circRNA circRNA	circRNA microarray circRNA microarray	MACO mouse model AIS patients	Aqueous humor Plasma	Co-regulation of retinal neurodegeneration and cerebral neurodegeneration Mitophagy Regulation of hypoxia	High expression High expression	Becoming a potential biomarker for the diagnosis of cerebral and retinal nerve co-morbidities Rates of change of three RNAs in the first seven days as potential biomarkers for diagnosis and	[70, 124]
circPDS5B	circRNA				Cell proliferation Cell division			
circCDC14A	circRNA				Cell proliferation Cell division Cilium assembly Peptidyl-tyrosine dephosphorylation		prediction of stroke outcome	
circSCMH1 circHECTD1	circRNA circRNA	circRNA microarray circRNA microarray	AIS patients IS patients	Plasma Plasma	Regulation of the neuronal plasticity, glial activation, and peripheral immune cell infiltration Regulation of the astrocyte activation	Low expression High expression	To evaluate the prognosis of stroke patients, and can be used as a therapeutic target to improve the prognosis Its coupling reaction provides translational evidence for IS mechanism as a new potential therapeutic target	[125, 126]
Hsa_circRNA_0003574	circRNA	ceRNA microarray	Patients with IS caused by ICAS	Whole peripheral blood	Involved in wnt/β-catenin pathway	High expression	Novel biomarkers and potential therapeutic targets for ischemic stroke	[127]
SNHG15 lncRNA-ENST00000568243	lncRNAs lncRNA	lncRNA microarray LncRNA microarray	AIS patients IS patients	Peripheral blood mononuclear cells Whole peripheral blood	Inhibit peripheral inflammatory responses Regulation of neuron death Activation of MAPKK activity Positive regulation of apoptotic process Protein kinase activity	High expression High expression	Being a negative regulator of inflammation has the potential to be a new potential therapeutic target for IS For the diagnosis of IS	[134, 135]
miR-125a-5p, miR-125b-5p, miR-143-3p	microRNA	RNA-seq	AIS patients	Serum	May be associated with platelet aggregation or thrombosis	High expression	For the diagnosis of AIS	[138]
Hsa-miR-548c-5p	miRNA	RNA-seq	TIA and AIS patients	Serum	Modulation of circulation and vascularity	It is highly expressed in AIS patients compared with TIA patients	Used to identify AIS and TIA	[139]

### Proteomic patterns

Body fluid proteomics is a discipline that studies the composition and function of proteins in body fluids. Proteins are important functional molecules in living organisms, involved in cell structure and function, and play important roles in the organism such as regulating physiological processes and transmitting signals. The core goal of body fluid proteomics in IS is to reveal protein expression and functional abnormalities that are closely related to the development of IS by analyzing and interpreting protein composition and its changes in body fluids. Currently, proteomics is a kind of omics with the most research references and the widest practice. In this field, researchers have found many biomarkers involved in physical and pathological responses and attached to IS authentication diagnosis, process and prognosis prediction, personalized therapeutic targets, etc.

IS proteomics studies use multiple body fluids as samples, including blood, serum, plasma, CSF, urine, and saliva. Blood and its components (serum and plasma) are commonly used samples for proteomics research, and also significant testing fluid for IS diagnosis. This article summarized 36 proteins revealed to be biomarkers for IS in blood, serum, and plasma. These biomarkers exit in blood or its’ components, or in both and other types of fluids in the meantime. Among these biomarkers discovered in IS situations, there are 16 proteins demonstrated to increase, 7 proteins expressing lower, and 13 proteins found significant differences in expressions. Variations in protein expressions were associated with IS pathological mechanism and process, because of which they become biomarkers for IS predicted diagnosis, authentication of stroke types, and personalized therapeutic targets. First, for the function of prediction, it is reported that elevated S-100 can be used as a tool for peripheral marker to predict ischemic focal brain injury [141]. Similarly, the protein group’s early numerical changes provide predictors of AIS occurrence, which consists of NSE, MBP, and S100 β [142]. MMP-9, which was significantly associated with susceptibility to IS, can be used as an indicator to prevent and predict the onset of IS [143]. Besides, CKB, CMPK, hemoglobin, fibronectin, and antimicrobial protein REG3A were reported to be biomarkers for the prediction of IS diagnosis, treatment efficiency, and prognosis [30, 51, 144, 145]. Second, as for authentication of stroke types, it is published that apoproteins including apoA-I, A-II, B, C-I, C-II, C-III, D, E, H, and paraoxonase-1 were used to distinguish between ischemic and hemorrhagic stroke [146, 147]. S100-A9 was revealed to be a biomarker to differentiate between APIS and ANPIS [148, 149], and GFAP was a more sensitive marker for discriminating smaller luminal lesions from minor strokes in brain injury [150]. Third, some proteins in blood can be regarded as target for personalized therapy or indicators to assess the disease process. For example, S-100 B release may be a useful tool for monitoring and evaluating therapeutic interventions, a single of which can be used as a surrogate marker for early and adequate MCA/M1 recanalization [151, 152]. Some proteins expressing differently during the disease have been straightly regarded as targets for pharmacological intervention and other personalized treatment, such as SAA 1, prekallikrein, SWAP70, thymidine phosphorylase, Hsp70-8, Hsp70-2, SAHH2, SCARA5, and MMP family proteins (including MMP-1, MMP-8, and MMP-12) [30, 41, 48, 49, 153, 154].

Besides blood and its components, IS proteomics research has involved many other body fluids. First, CSF, which directly responds to the physical or pathological situations in the brain, is an important sample. There are many biomarkers found in CSF associated with IS, besides protein S-100, prekallikrein, MMP-12, SWAP70, CKB, CMPK, and SAHH2 mentioned above, circulating H-FABP extracted in CSF elevated after IS, through which they put forward it could be a potential biomarker for predicting brain injury [155]. Second, some people tested a group of proteins in urine, including ACP2, PLD3, HLA-C, GGH, CALML3, and IL2RB. This proteomic pattern is composed of six urinary proteins that show the best IS prediction accuracy, and the combination can serve as a potential diagnostic marker and therapeutic target and can be used to differentiate between symptomatic and asymptomatic CAS with high sensitivity and specificity [61]. What’s more, some people put forward that saliva is also a potential fluid with rich protein content for the discovery of IS biomarkers. As is reported, salivary xanthine oxidase, which participates in neuronal oxidative stress in IS, was found to be significantly differentiated between ischemic and control and hemorrhagic stroke groups. This finding revealed that salivary xanthine oxidase can be a biomarker for authentic diagnosis and prediction of prognosis [64]. Similarly, salivary cortisol is demonstrated to be involved in the pathological process of hippocampus damage and is significantly expressed higher in MCI with postoperative IS than in controls, indicating salivary cortisol levels can be a predictor of MCI occurrence [63].

In terms of proteins, there are some typical proteins involved in IS pathological responses, such as S-100 series proteins, apoproteins, and MMPs. First, s-100 series protein is the largest subgroup of calcium transporter proteins, which IS has also been shown to be engaged in the structural differentiation of the cytoskeleton and Ca^2+^-dependent cell information processing [156–158]. S-100 proteins are associated with many chronic diseases, such as cancer, cardiovascular disease, and especially cerebral nervous disease like IS. Variations shown during the process of IS closely relate to damage to astrocytes and inflammatory responses [156, 157, 159], as markers of brain tissue damage and progression of IS, through which people speculate about the extent of brain tissue damage and judge the progression of IS. Second, apoproteins, which participated in lipid metabolism, are also obviously associated with the progression of IS, similar to other cardiovascular diseases like atherosclerosis and hyperlipidemia. Variations of apoproteins differentiate in subtypes of stroke significantly, perhaps because of the different types of lipoproteins they transfer. Therefore, apoproteins can be used to distinguish types of stroke with considered accuracy [146, 147, 159]. Third, MMPs play a main role in IS biomarker discovery. MMPs are a kind of protein hydrolase, whose primary role is organizational remodeling, and participate in many responses such as cellular remodeling, angiogenesis, and damage recovery. MMPs are a signal that tissues are repairing themselves after the occurrence of disease. Therefore, the variation of MMPs indicates the onset or progression of IS, which can be used for predictive diagnosis [143], and also, MMPs can serve as potential therapeutic targets for personalized or pharmacological intervention [48]. Thereby, these three proteins are important components of IS proteomics research and have the potential for further development in the future.

In summary, body fluid proteomics has a wide application in multiomic studies of IS, contributing to the advancement of diagnosis, treatment, and prevention of IS. Table 4 collates protein biomarkers that may be beneficial for IS prevention, prediction, and personalized treatment.

**Table 4:** Typical proteomics body fluid biomarkers of IS. *MCA*, middle cerebral artery; *IS*, ischemic stroke; *ANPIS*, acute non-progressive ischemic stroke; *APIS*, acute progressive ischemic stroke; *ICH*, intracerebral hemorrhage; *DIA*, data-independent acquisition; *CAS*, carotid artery stenosis; *MCAO*, middle cerebral artery occlusion; *SRM*, aptamer-based proteomic assay; *MR*, mendelian randomization; *MCI*, mild cognitive impairment

Protein	Body fluid	Technique	Sample	Variations	Function/potential function	Application in PPPM	References
Thymidine phosphorylase	Plasma	LC–MS	AIS patients	Significantly increased levels in stroke patients	Inhibition of collagen and ADP-induced platelet aggregation	TYMP-4 may be a personalized therapeutic target for stroke	[30]
Hsp70-8, Hsp70-2					Cooperating with anti-apoptotic factors and reducing the area of cell death in the brain	Could be a target for personalized stroke therapy	
Fibronectin	Plasma	LC–MS		Positive correlation between and risk of hemorrhagic transformation after thrombolytic therapy for ischemic stroke	Involved in thrombosis and inflammatory response	Markers of blood–brain barrier damage that predict treatment efficacy and prognosis	
SCARA5	Blood	MR, multiplex assays, GWAS	Arge artery atherosclerosis, cardioembolic stroke, and small artery occlusion stroke patients	Associated with reduced risk of ischemic stroke	Novel protective mediators for cardioembolic stroke with reduced risk of subarachnoid hemorrhage	Promising targets for the treatment of cardiogenic embolic stroke	[41]
prekallikrein, MMP-12 and SWAP70	Plasma and CSF	MR analysis, PPI	/	Elevated prekallikrein, MMP-12, and SWAP70 are significantly associated with reduced IS risk	/	MMP-12 and prokinetic peptide-releasing enzyme may serve as personalized therapeutic targets for pharmacological intervention in IS	[49]
Antimicrobial protein REG3A	Plasma	MS	Patients undergoing mechanical thrombectomy for large-vessel occlusion stroke	Systemic REG3A levels positively correlate with the inflammatory proteins interleukin IL 6 and IL 17 C and NIHSS scores corresponding to moderate and moderate-severe neurological deficits	Involved in inflammatory responses and cell survival processes	Predicting functional recovery within hours of stroke	[51]
ACP2, PLD3, HLA-C, GGH, CALML3, and IL2RB	Urine	DIA	Patients with CAS	Proteome composed of 6 urinary proteins shows the best IS prediction accuracy	/	The combination can serve as a potential diagnostic marker and therapeutic target and can be used to differentiate between symptomatic and asymptomatic CAS with high sensitivity and specificity	[61]
Salivary cortisol	Saliva	Electrochemiluminescence immunoassay	patients with MCI after cerebral ischemic stroke (CIS) and 80 CIS patients without MIC	Salivary cortisol levels were significantly higher in MCI with postoperative IS than in controls	Participates in the pathological process of hippocampus damage	Salivary cortisol levels can be a predictor of MCI occurrence	[63]
Salivary xanthine oxidase	Saliva	Fluorimetry	IS patients and hemorrhagic stroke patients	Significant differences between ischemic and control and hemorrhagic stroke groups	Participates in neuronal oxidative stress in ischemic stroke	Potential biomarkers for differential diagnosis of IS and prediction of prognosis	[64]
S-100	Cerebrospinal fluid and serum	Two-site radioimmuno assay	Patients with an acute infarction in the territory of the MCA	Elevated S-100 compared to controls	Presumably related to a combination of necrotic glial cell leakage and damage to the cerebral blood barrier	Serum S-100 can be used as a peripheral marker to predict ischemic focal brain injury	[141]
NSE, MBP, S100 β	Serum	ELISA	AIS patients	Elevated in the first 24 h after stroke	NSE is a dimeric isoenzyme of the glycolytic enzyme enolase. Myelin basic protein (MBP) 1–5 is a rich myelin protein lipid produced by oligodendrocytes. S100β is a cytoplasmic calcium-binding protein	Its early numerical changes provide predictors of AIS occurrence	[142]
MMP-9	Peripheral blood	SNP-SNP interaction	IS patients	Significantly associated with susceptibility to IS	Strong association with intracranial aneurysms, atherosclerosis, ischemic brain injury, and other diseases	Can be used as an indicator to prevent and predict the onset of IS	[143]
MMP-1,MMP-8,MMP-12	Serum and plasma	Mendelian randomization approach	/	There was a causal relationship between lower serum MMP-12 levels and risk of ischemic stroke, between lower serum MMP-1 and MMP-12 levels and risk of large-artery stroke, and between higher serum MMP-8 levels and risk of lacunar stroke		Potential as a biomarker and therapeutic target for stroke risk	[48]
CKB, CMPK	CSF and human blood	SOMA scan	MACO mouse model	CKB and CMPK levels were higher in IS patients than in the acute phase	Catalyzing the reversible transfer of phosphate	Potential to predict and diagnose IS	[144]
Hemoglobin	Serum	MALDI-TOF MS	AIS patients	Significant difference in peak hemoglobin alpha and beta chain expression between stroke and control groups	Oxygen-carrying	As a possible biomarker for IS diagnosis	[145]
ApoA-I, A-II, B, C-I, C-II, C-III, D, E, H	Plasma	SRM assay	Acute stroke patients	The combination of apoC-III and apoA-I accurately differentiated ischemic and hemorrhagic groups; apoC-III and apoC-I optimally differentiated ischemic and control groups	Participates in lipoprotein metabolism	ApoA-I, AII, B, C-I, C-II, C-III, D, E, and H are used to distinguish between ischemic and hemorrhagic stroke	[146]
Apo A-1, paraoxonase-1	Plasma	Multiplex assays	IS patients and ICH cases	Apo A-I was lower in IS than in controls, and Paraoxonase-1 was lower in the IS case group than in the cerebral hemorrhage and control groups	Apo A-1 is the major lipoprotein associated with HDL; Paraoxonase-1 hydrolyzes oxyphosphate metabolites and prevents vascular disease by metabolizing oxidized lipids	Distinguishing between hemorrhagic and ischemic stroke	[147]
S100-A9	Serum	LC–MS/MS	APIS and ANPIS patients	Elevated after stroke and ischemia	Involved in physiological activities in response to stress, inflammation, and complement activation	Potential biomarker to differentiate between APIS and ANPIS	[148, 149]
GFAP, S-100B	Serum	Two-site immunoluminometric assay	IS patients	Sustained increase from admission to day 4 post-stroke	Involvement in differentiation of cytoskeletal structures and Ca2 + -dependent cellular information processing reflects damage to astrocytes	Postischemic GFAP and S-100 B release may be a useful tool for monitoring and evaluating therapeutic interventions; GFAP is a more sensitive marker for discriminating smaller luminal lesions from minor strokes in brain injury	[150]
GFAP		In-house enzyme-linked immunoassay			It is speculated that GFAP may play an important role in the control of glutamine metabolism after stroke		
S100 B	Serum	Two-site immunoluminometric assay	MCA/M1 occlusion patients	Recirculation was associated with significantly lower mean S100B levels compared to no recirculation	Markers of brain tissue damage reflecting astrocyte damage	A single S100 B value serves as a surrogate marker for early and adequate MCA/M1 recanalization	[151]
			Patients with acute nonlacunar middle cerebral artery infarction	Single measurements of S100 B serum concentrations at 48 and 72 h after stroke onset highly correlate with long-term prognosis and infarct volume		Single S100 B values obtained at 48 and 72 h after stroke onset provided excellent predictive value for functional outcome and infarct volume in nonlacunar middle cerebral artery infarction	[152]
SAA 1	Serum	DCF fluorescence analysis	Mouse stroke model	Elevated after stroke and ischemia	Mediates systemic inflammatory response	Potential therapeutic target for IS	[153]
SAHH2	Brain tissue and blood	MS/MS	Post-mortem brain slices from ischemic stroke patients	Low levels of circulating SAHH2 at 24 and 48 h after IS onset are strongly associated with improved neurological functioning	Inhibition of Ca2 + ion transport proteins	Potential as biomarkers for neuroimprovement prognosis, cerebral ischemia representation, or therapeutic targets	[154]
H-FABP	CSF	2DE-MS	Post-mortem cerebrospinal fluid samples	Cerebrospinal fluid FABP elevated after stroke; circulating H-FABP elevated after IS	Involved in the storage and metabolism of long-chain fatty acids	Potential Biomarkers for Predicting Brain Injury	[155]
ApoC-I, ApoC-III	Plasma	SELDI-MS	Stroke patients	Significant difference in ELISA test levels between hemorrhagic and ischemic for ApoC-I and ApoC-III	Involved in triglyceride metabolism	ApoC-I and ApoC-III are plasma markers for differentiating ischemic and hemorrhagic stroke	[159]

### Metabolomic patterns

Metabolomics is a newly emerging omics study in recent years. Body fluid metabolomics of IS studies the changes of metabolites that are closely related to the pathogenesis and pathophysiological process of IS. As a bridge connecting various organs and tissues of the human body, body fluids contain metabolites such as amino acids and lipids that can often reflect physiological or pathological changes in the human body. Therefore, an in-depth study of body fluid metabolomics is of great significance in revealing the pathophysiological process of IS, discovering biomarkers, evaluating therapeutic effects, and guiding drug development.

The main technique of metabolomics is MS, and for different research features, metabolomics is divided into targeted and untargeted types. In some targeted metabolomics studies, researchers extracted metabolites in different fluids that potentially vary during the process of IS disease, and intend to prove the ability of them to be biomarkers. For example, some people discovered the essential fatty acid in plasma, linoleic acid, whose high level is significantly associated with the reduced risk of total CVD, cardiovascular mortality, and IS, and its increase in dietary improvement leads to the prevention of IS and other cardiovascular diseases [50]. Besides, some researchers found some metabolites in plasma altered in the pathology of stroke, and regarded them as biomarkers for prediction and diagnosis. It is reported that high levels of succinic acid are related to an increased risk of cardiac stroke, atrial dysfunction, and left atrial enlargement; thus, changes in succinic acid levels contribute to risk prediction of CE stroke and atrial dysfunction [160]. There is also a group of metabolites including glucosylceramide (38:2), triacylglycerol (56:5), phosphatidylethanolamine (35:2), and free fatty acid (16:1) involved in lipid and energy metabolism, which found that a combination of which achieves better AUC values, sensitivity, and specificity in lacunar infarction can be novel biomarker for disease diagnosis [161]. Moreover, as is proved in target metabolomics research with a mouse model, researchers found that levels of lipids like ceramide 42:1 and sphingomyelin 36:0 extracted in the brain and plasma of mice were significantly higher in stroke group compared to the control, and suggested that the lipids contribute to prediction and diagnosis of stroke [162].

Untargeted metabolomics studies are the main type of the documented IS metabolomics research, which not only discovered many stroke biomarkers but also covered different kinds of body fluids. First, blood as the main body fluid sample in science research and clinical practice is not more utilized in metabolomics than its components, plasma, and serum. The reason might be because the whole blood contains many kinds of blood cells, which interferes with metabolomics analysis. Thus, besides the targeted researches described above, many metabolites were obtained from plasma and serum as biomarkers of IS for predictive diagnosis, prevention, and personalized therapy. Through GC/LC–MS, one study selected serine, isoleucine, betaine, PC(5:0/5:0), and LysoPE (18:2) frequently involved in metabolism responses in serum and found them discriminate accurately between AIS and control samples, defining these five metabolites as novel serum biomarker models with potential predictive and diagnostic value [163]. Similarly, some people found uric acid, sphinganine, and adrenoyl ethanolamide elevated in IS patients, and these three metabolites contribute to the pathomechanisms of IS, which are used as a potential biomarker for predicting the pathogenesis of IS [164]. Indeed, many studies focus on the predictive function of serum or plasma metabolites. For example, LysoPC (20:4) and LysoPC (16:0) were regarded as biomarkers for IS prediction [165], and tyrosine, lactate, and tryptophan were revealed to be not only the predictive markers but potential therapeutic targets [166]. What’s more, some people utilized mouse models and combined studies of plasma and CSF and discovered changes in branched-chain amino acid levels contribute to outcome prediction in IS and hold promise as a new therapeutic target [167].

Urine is also a considerable type of body fluid for IS biomarker discovery. With the new technique 1H-NMR, one study reported that many metabolites contained in urine and plasma could be regarded as biomarkers for cerebral infarctions. Citrate, hippurate, and glycine changing in urine and lactic, pyruvic, glycolic, formate, glutamine, and methanol altering in plasma were revealed to be associated with stroke, and their combination was regarded as an independent risk factor for IS, especially for SVO in lacunar cerebral infarction, and contributed to the diagnosis and prognosis of IS [58]. Similarly, ones tested the varying metabolites, glycine, and acetylcarnitine in the urine of AIS patients, and revealed a longitudinal demonstration of pathophysiological processes of amino acid metabolism in IS, which contributes to the discovery of new therapeutic targets [62]. Moreover, metabolomics was also carried out in other body fluids, such as tears, for IS biomarker discovery. Many metabolites were found to change in tears of diabetic retinopathy patients, which includes 5-aminovaleric acid, glycolithocholic acid sulfate, ceramides (d18:1/18:0(OH)), xanthine, lysophosphatidyl-cholines aC24:0, aC26:0, aC26:1, phosphatidyl-cholines ae C30:0, aeC38:0, aeC40:4, and triacylglycerides (16:1_36:1) [11]. This study confirmed the overlapping course of IS and diabetic retinopathy, and these metabolites were potential biomarkers for IS as well as diabetic retinopathy.

Thereby, metabolomics can be carried out in variable types of body fluids and contributes a lot to discovering new biomarkers for IS predictive diagnosis, prevention, improvement of prognosis, and new therapeutic targets. Table 5 collates metabolite biomarkers for IS prevention, prediction, and personalized treatment.

**Table 5:** Typical metabolomics body fluid biomarkers of IS. *TBI*, traumatic brain injury; *CE stroke*, cardioembolic stroke; *NP/RP 2D LC-QToF/MS*, normal-phase/reversed-phase two-dimensional liquid chromatography-quadrupole time-of-flight mass spectrometry; *LysoP*, lysophosphatidylcholine; *SVO*, small-vessel occlusion

Metabolite	Body fluid	Approach	Targeted/untargeted	Sample	Function	Findings	Application in PPPM	Reference
5-Aminovaleric acid, Glycolithocholic acid sulfate, Ceramides (d18:1/18:0(OH)), Xanthine, Lysophosphatidyl-cholines aC24:0, aC26:0, aC26:1 Phosphatidyl-cholines ae C30:0, aeC38:0, aeC40:4 Triacylglycerides (16:1_36:1)	Tear	MS	Untargeted	Stroke patients	Confirmation of the overlapping course of IS and diabetic retinopathy	The same level differences were found in diabetic retinopathy and IS	3PM potential biomarkers for becoming a combined risk for IS as well as diabetic retinopathy	[11]
Linoleic acid	Plasma	GC/MS	Targeted	/	Essential fatty acids that cannot be synthesized by the body and are the main dietary polyunsaturated fatty acids	High levels of LA significantly associated with reduced risk of total CVD, cardiovascular mortality, and ischemic stroke	Providing a basis for dietary prevention of IS and other cardiovascular diseases	[50]
Citrate, hippurate, and glycine	Urine	1H-NMR	Untargeted	Patients with cerebral infarctions	Association of anaerobic glycolysis, folate deficiency, and hyperhomocysteinemia	Reduced urinary levels of citrate, hippurate, and glycine in stroke patients	It is an independent risk factor for IS, especially for SVO in lacunar cerebral infarction, and contributes to the diagnosis and prognosis of IS	[58]
Lactic, pyruvic, glycolic, formate. Glutamine and methanol	Plasma					Increased plasma excretion of lactate, pyruvate, glycolic acid, and formate acid and decreased excretion of glutamine and methanol in stroke patients		
Glycine and Acetylcarnitine	Urine	LC–MS	Untargeted	Stroke patients in acute (72 h) and chronic (3–5.2 months) stage	Involved in amino acid and fatty acid metabolism, thought to be a source of oxidative stress	Glycine concentrations decrease and acetylcarnitine increases in the acute phase of IS	Longitudinal demonstration of pathophysiological processes of amino acid metabolism in IS contributes to the discovery of new therapeutic targets	[62]
Succinate	Plasma	LC-QQQ/MS	Targeted	Acute stroke patients	Intermediates of the TCA cycle	High levels of succinic acid associated with increased risk of cardiac stroke, atrial dysfunction, and left atrial enlargement	Changes in its levels contribute to risk prediction of CE stroke and atrial dysfunction	[160]
Glucosylceramide(38:2), triacylglycerol(56:5),phosphatidylethanolamine (35:2), and free fatty acid(16:1)	Plasma	NP/RP 2D LC-QTOF/MS	Targeted	Lacunar infarction patients	Involved in lipid and energy metabolism	Combination of four metabolites achieves better AUC values, sensitivity, and specificity	Defining four metabolites as novel biomarkers for the diagnosis of lacunar infarction	[161]
Ceramide 42:1 Sphingomyelin 36:0	Brain tissue and plasma	LC–MS	Targeted	MACO and TBI mouse model	/	Compared to the control group, lipid levels were significantly higher in the stroke group, especially Ceramide 42:1 and Sphingomyelin 36:0	Contributes to the prediction and diagnosis of AIS	[162]
Serine, isoleucine, betaine, PC (5:0/5:0), and LysoPE (18:2)	Serum	GC–MS, LC–MS	Untargeted	AIS patients	Involved in amino acid, choline, and phospholipid metabolism	Five metabolites discriminate accurately between AIS and control samples	Defining these five metabolites as novel serum biomarker models with potential predictive and diagnostic value	[163]
Uric acid, sphinganine, and adrenoyl ethanolamide	Serum	UPLC/QTOF MS/MS	Untargeted	IS patients	Involves purine metabolism, sphingolipid metabolism, and fatty acid metabolism	Three metabolites were significantly elevated in IS relative to controls	Contributes to in-depth investigation of the pathomechanisms of IS and is a potential biomarker for predicting the pathogenesis of IS	[164]
LysoPC (20:4) and LysoPC (16:0)	Plasma	MS/MS	Untargeted	TIA patients	Plays a role in inflammation in ischemic recurrence or tolerance and has some anti-inflammatory potential	Low concentrations of specific LysoPC [16:0] and LysoPC [20:4] were significantly associated with IS recurrence	Potential biomarkers for IS prediction	[165]
Tyrosine, lactate, and tryptophan	Serum	GC–MS	Untargeted	AIS patients	Involved in amino acid metabolism	The overall percentage of predicted IS correction for the three amino acids was 91.7%	Potential prediction biomarkers and therapeutic targets for disease in AIS	[166]
Branched-chain amino acids	Plasma and CSF	LC–MS/MS	Untargeted	Ischemic stroke mouse model and acute stroke patients	Plays a role in protein metabolism and catabolic energy metabolism	Plasma BCAAs were significantly reduced compared to control	Changes in its levels contribute to outcome prediction in IS and hold promise as a new therapeutic target	[167]

## Conclusions and expert recommendations in the framework of 3PM

### Conclusions

The above highlighted scientific evidence supports the feasibility and credibility of both working hypotheses A and B presented in this article, namely “*A. Multi‑factorial systemic risks of IS are preventable making a holistic 3PM approach in primary care to the most cost-effective IS management*” and “*B. Body fluid molecular patterns are instrumental for holistic 3PM-guided population screening, health risk assessment, targeted prevention, and therapy monitoring in primary and secondary care of IS*”.

**Fig. 4:**
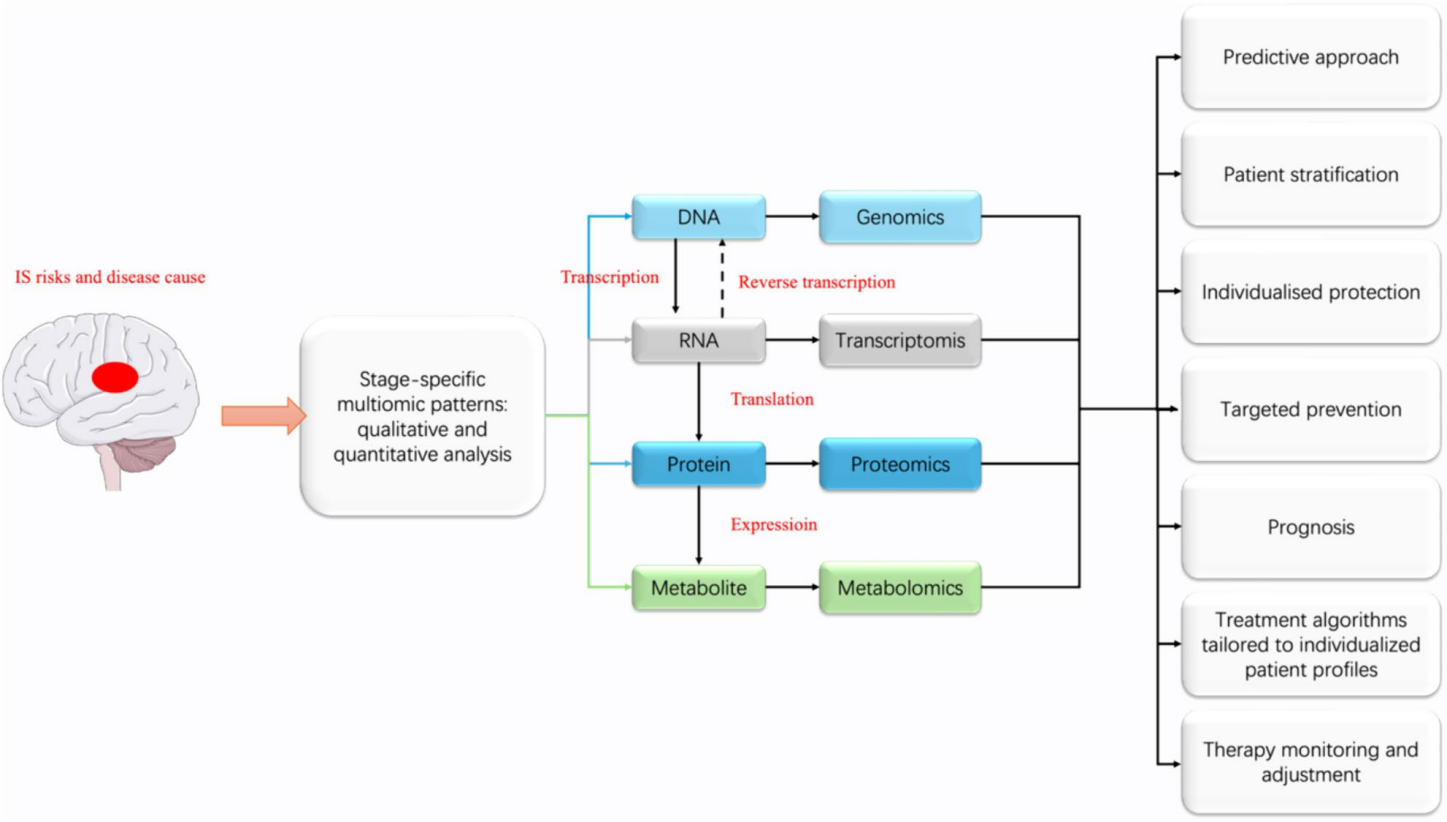
Body fluid multiomics is instrumental for IS prediction, prognosis, targeted prevention, treatments tailored to individualized patient profiles, therapy monitoring, and adjustment

During this whole paper, we’ve been trying to prove the validity and value of these two hypotheses by collecting many literatures and integrating research associated before. There are many kinds of risk factors leading to IS but most of them are preventable, which reveals the importance of 3PM patterns in primary and secondary care. The form of the two kinds of healthcare will enter a new status with the instrumental tool, body fluid multiomics patterns, in the future. Body fluid multiomics is instrumental for IS prediction, prognosis, targeted prevention, treatments tailored to individualized patient profiles, therapy monitoring, and adjustment, which is described in Fig. 4. Biomarkers associated with IS will make the pathological mechanism, such as oxidative stress, ischemia refusion damage, and mitochondrial damage, more correct, and contribute to predictive diagnosis, risk factors prevention, personalized therapy, and improvement of prognosis.

The occurrence and development of IS involve a variety of pathophysiological changes [23, 27], and different omics methods reveal relevant information of different biological stages. Integrating the information of DNAs, RNAs, proteins, and metabolites obtained by different omics methods can define the landscape of molecular alterations in IS; in particular, specific molecular signatures can be detected in body fluids, which will provide new insights into the etiology, pathology, therapeutic targets, and related biomarkers of IS, thereby advancing the clinical application of 3PM in patients with IS [168].

**Fig. 5:**
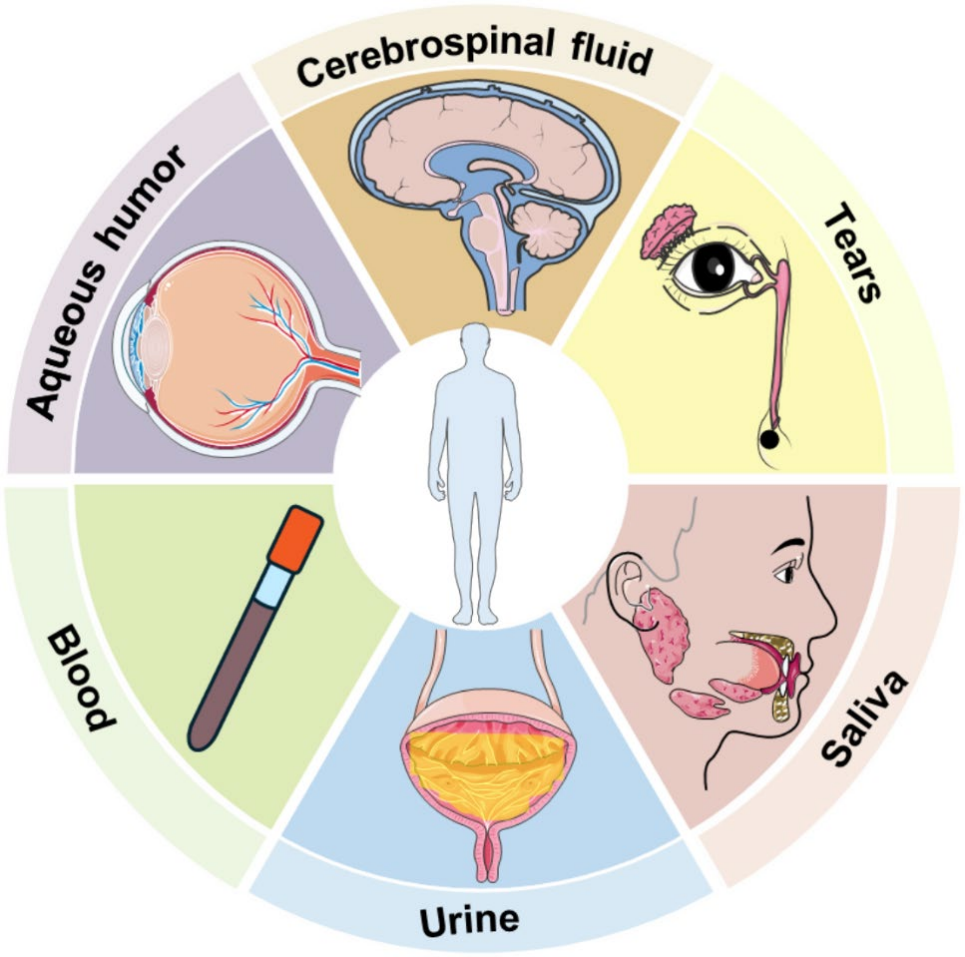
Targeted body fluids for multiomics analysis in ischemic stroke

The advantages and disadvantages of individual body fluids are thoroughly analyzed throughout the paper and summarized in Fig. 5 (Box 1). For example, multiomics based on a minimally invasive approach utilizing blood and its components (plasma and serum) is strongly recommended for a holistic real-time monitoring, due to the particularly high level of dynamics of the blood as a body system. CSF can reflect brain conditions directly and contains many kinds of biomoleculars reflecting IS more correctly, but as a body fluid with serious invasive, it is difficult to be one of the assessed body fluids in everyday healthcare. Aqueous humor is similar to CSF; the invasive operation and limited samples make it hard to spread it as everyday ones. However, for science research, these two body fluids are still worth studying for physical and pathological mechanisms. Besides, as is explained in this paper, some non-invasive body fluids, such as urine, saliva, and tears, definitely own plenty of space for research of brain diseases, especially IS. Urine and saliva contain a lot of biomacromoleculars in similar way, but also with the same defect that they are less stable for examination. In contrast, tear fluid as a more stable system is more appropriate for a non-invasive and patient-friendly holistic approach appropriate for health risk assessment and innovative screening programs in cost-effective IS management.

Box 1 Clinically and technologically relevant advantages vs. disadvantages of targeting specific sorts of the body fluid for IS multiomic analysis.

**Table:** 

	Advantages	Disadvantages	References
Cerebrospinal fluid	Directly reflecting nerve center conditions and rich in biomolecules	Invasive sampling; high risk of contamination; significant individual differences	[169, 170]
Tears	Non-invasive, technologically simple sampling; specific molecular patterns are stable specifically in basal tears and reflect systemic effects relevant for IS risks, disease development and progression, and patient stratification; cost-effective and patient-friendly procedure appropriate for a holistic approach and population screening in primary and secondary care	Data interpretation strongly depends on the robustness of the corresponding database	[11, 17, 20, 74]
Aqueous humor	Relevant for eye-associated pathologies	Invasive sampling; limited applicability for a holistic approach	[171]
Saliva	Non-invasive, technologically simple sampling; cost-effective and patient-friendly procedure appropriate for a holistic approach and population screening in primary and secondary care	Low specificity; many irrelevant confounding factors	[172, 173]
Blood	Large number of biomolecules; minimally invasive sampling; real-time monitoring	Complexity; many confounding factors; problematic data interpretation due to high dynamics of molecular profiles	[174]
Urine	Non-invasive sampling; relevant for whole body metabolic status	Low specificity; diluted relevant patterns; problematic quantification	[175]

### Expert recommendations

Although several studies identified molecular patterns specific for IS in body fluids, none of these approaches has yet been incorporated into IS treatment guidelines. Below, we emphasize aspects essential to promote practical implementation of highlighted achievements in 3PM-guided IS management.

(i) Validate potential biomarker information. It is particularly important to validate potential biomarker information in large independent samples, especially across different ethnicities. Validation in large independent samples is lacking for many potential biomarkers identified in the body fluids of IS patients and animal models. The source of omics data often involves comparing diseased populations with reference populations, and it is necessary to validate these findings with a sufficient number of matched individuals to minimize confounding factors. Additionally, validation across different racial/ethnic groups is essential to determine applicability among diverse ancestral populations.
(ii) Standardize body fluid collection time. After IS occurs, cells respond to the corresponding pathological processes through gene expression regulation, indicating that the biological processes within the patient’s body are constantly changing during different time periods after cerebral ischemia. Additionally, intervention treatments for patients also cause pathological and physiological changes. Standardizing the collection time of bodily fluids can help eliminate deviations caused by the dynamic evolution process of the disease to a certain extent.
(iii) Combine in vitro and in vivo molecular research to determine the driving factors of IS. It is often challenging to collect omics data at specific time points, so extracting causal information between various omics data and diseases is a common challenge in the current field of omics [168]. In the context of relevant omics data, experimental animal models or cell models can be used to demonstrate causal associations between specific changes and IS.
(iv) Explore new approaches to obtain genetic information of the nervous system. The nucleic acid information obtained through bodily fluids often primarily comes from cells in peripheral blood. Due to the different expression patterns of nervous system genes in peripheral blood compared to the brain or nerves, and the difficulty in obtaining brain tissue for analysis in patients with IS (immune system) disorders, it may be necessary to employ new methods such as inducing pluripotent stem cells from patients in vitro to differentiate into neurons and obtain nucleic acid information from neurons and brain tissue.
(v) Perform accurate phenotyping. The etiological subtyping of IS is mainly based on the Trial of Org 10,172 Acute Stroke Treatment (TOAST) and Causative Classification of Stroke (CCS) systems [169, 170], with the ongoing debate regarding their consistency [171]. When studying genetic factors and corresponding functional outcomes in complex diseases such as IS, it is crucial to apply the same subtype classification system and accurately characterize the phenotype.
(vi) Integrate multiple omics data to explore a combined diagnostic and therapeutic model for IS. Because IS is a complex multifactorial disease involving potential alterations in multiple molecular pathways simultaneously, a specific molecular alteration identified by a single omics technique may not be sufficient to influence the pathophysiological course of IS. By integrating multiple omics information related to IS, one can uncover interconnected molecular mechanisms within biological processes [176, 177], thereby facilitating the development of a combined diagnostic and therapeutic approach for IS.
(vii) Resolve technological and processing issues. There are still many unresolved problems in the face of somatic multiomics for IS today [178, 179]. For example, (A) technical limitations: despite the continuous development of multiomics technologies (e.g., genomics, transcriptomics, proteomics, metabolomics), there are still some technical limitations such as sample processing, data storage, and analysis methods that may not be sufficiently mature or standardized, which may lead to bias or inconsistent results. (B) Complexity of data integration: Multiomics data come from different biomolecular levels, and their integration and analysis require complex bioinformatics tools and algorithms. Data integration not only involves conversion and standardization of data formats, but also needs to consider the interactions and associations between different histological data, which makes the analysis process complex and error prone. (C) Sample differences and individual differences: The molecular composition of body fluid samples may be affected by a variety of factors, such as age, gender, genetic background, and lifestyle. These differences may lead to incomparability or bias in research results. In addition, inter-individual variability may make it difficult to generalize the findings of multiomic studies. (D) Disease complexity and dynamics: Many diseases are complex and dynamic, involving changes at multiple biomolecular levels. While multiomic studies of body fluids can provide a wealth of information, it may be difficult to fully capture the complexity and dynamics of disease. Consequently, conclusions based on multiomics data may not be sufficiently accurate or comprehensive. (E) Ethical and privacy issues: Multiomic studies involve a large amount of personal biological information, which raises concerns about data privacy and ethics. How to fully utilize these data for analysis and research while protecting individual privacy is an urgent issue to be addressed.

## Supplementary Information

Below is the link to the electronic supplementary material.

Supplementary Material

## Data Availability

All the data used in this study are presented in the article.

## Abbreviations

2-DE: Two-dimensional gel electrophoresis
3PM: Predictive, preventive, and personalized medicine
ABI: Ankle-brachial index
AD: Alzheimer’s disease
AIS: Acute ischemic stroke
ANPIS: Acute non-progressive ischemic stroke
APIS: Acute progressive ischemic stroke
BAIAP 2: Brain-specific angiogenesis inhibitory factor 1-associated protein 2
BPD: Borderline personality disorder
CAS: Carotid artery stenosis
CCS: Causative classification of stroke
CE stroke: Cardioembolic stroke
cIMT: Carotid intima-media thickness
circRNA: Circulation RNA
CSF: Cerebrospinal fluid
DIA: Data-independent acquisition
DM: Diabetes mellitus
eccDNA: Extrachromosomal circular DNA
EWAS: Epigenome-wide association studies
FGF21: Fibroblast growth factor 21
FSP: Flammer syndrome phenotype
GC-MS: Gas chromatography-mass spectrometry
GWAS: Genome-wide association studies
HuR: Human antigen R
ICAS: Intracranial atherosclerotic stenosis
ICH: Intracerebral hemorrhage
IS: Ischemic stroke
iTRAQ: Isotope tagging for relative and absolute quantification
LAA: Large-artery atherosclerotic stroke.
LC-MS: Chromatography-mass spectrometry
LD: Linkage disequilibrium
lncRNAs: Long non-coding RNAs
MACO: Middle cerebral artery occlusion
MCA: Middle cerebral artery
MCAO: Middle cerebral artery occlusion
MCI: Mild cognitive impairment
MCI: Mild cognitive impedance
MeDIP: Methylated DNA immunoprecipitation
MeDIP-sequq: Methylated DNA immunoprecipitation sequencing
MIP-1α: Macrophage inflammatory protein-1 alpha
miRNA: MicroRNAs
MR: Magnetic resonance
MR: Mendelian randomization
MS: Mass spectrometry
PAGln: Phenylacetylglutamine
PDR: Proliferative diabetic retinopathy
PPPM: Predictive preventive personalized medicine
RNA: Ribonucleic acid
RRBS: Reduced representative bisulfite
rtPA: Recombinant tissue-type plasminogen activator
SMRBS: Single-molecule, real-time bisulfite sequencing
SMRT-BS: Bisulfite sequencing
TBI: Traumatic brain injury
TIA: Transient ischemic attack
TOAST: Trial of Org 10172 Acute Stroke Treatment
UA: Uric acid
WGBS: Whole genome bisulfite sequencing
XO: Xanthine oxidase

## Code availability

All protein and gene accession codes can be available in the Swiss-Prot and Genbank databases.
