# Supplementary material for: Body fluid multiomics in 3PM-guided ischemic stroke management: health risk assessment, targeted protection against health-to-disease transition, and cost-effective personalized approach are envisaged
Source: EPMA J. 2024 Aug 29;15(3):415–52. doi: 10.1007/s13167-024-00376-2 (PMC11371995; doi:10.1007/s13167-024-00376-2)
Supplement: Supplementary file 1 — Supplementary Material [file 13167_2024_376_MOESM1_ESM.docx]

**PPPM Innovation Highlights**

1. **Working hypotheses**

**Hypothesis A: Multi‑factorial systemic risks of IS are preventable making a holistic 3PM approach in primary care to the most cost-effective IS management**

**Hypothesis B: Body fluid molecular patterns are instrumental for holistic 3PM-guided population screening, health risk assessment, targeted prevention and therapy monitoring in primary and secondary care of IS**

1. **Innovation towards the predictive approach, targeted prevention and personalisation of medical services**

**Predictive approach**

Tear fluid as a more stable system is recommended for non-invasive and patient-friendly holistic approach appropriate for health risk assessment and innovative screening programs in cost-effective IS management.

**Targeted prevention**

Many independent studies demonstrated sufficient space for prevention interventions in the primary care of ischemic stroke defined as the most cost-effective protection of vulnerable subpopulations against health-to-disease transition. The paper details corresponding mechanisms and provides examples for utilizing body fluids for health risk assessment followed by mitigation measures and targeted prevention.

**Personalized medical services**

For example, multiomics based on minimally invasive approach utilizing blood and its components (plasma and serum) is recommended for real-time monitoring, due to particularly high level of dynamics of the blood as body system.

1. **How does the presented innovation go beyond the state of the art contributing to the paradigm shift from reactive medicine to PPPM?**

Although several studies identified molecular patterns specific for IS in body fluids, none of these approaches have yet been incorporated into IS treatment guidelines. Advantages and disadvantages of individual body fluids are thoroughly analyzed throughout the paper.
